# Impacts of Quinone Structure on Trade‐Offs Between Redox Potential and CO_2_ Binding Strength

**DOI:** 10.1002/cphc.202500472

**Published:** 2026-04-07

**Authors:** Jack S. Taylor, Alexander C. Forse, Alex J. W. Thom

**Affiliations:** ^1^ Yusuf Hamied Department of Chemistry University of Cambridge Cambridge CB2 1EW UK

**Keywords:** CO_2_ capture, electrochemistry, quinones

## Abstract

Quinones are well‐studied and promising candidates for redox‐active sorbents in electrochemical CO_2_ capture and storage (ECCS). Unfortunately, reactivity with O_2_ is a persisting issue in the use of quinones. While attempts have been made to tune the redox‐potentials of quinones to avoid this, quinones which are more tolerant to the presence of O_2_, are also generally less reactive toward CO_2_, that is, there is an inherent trade‐off. Building off of prior work on anthraquinones and fluorinated benzoquinones **[Bui et al., J.Phys.Chem.C., 126, 14 163‐14 172,2022]**, this article investigates how the trade‐off varies for a wider range of quinone types. No single unifying trade‐off is found; instead distinct quinone families follow distinct trade‐offs. A Hückel model is utilized to demonstrate how the change in aromaticity upon two‐electron reduction strongly influences the redox‐potential, and that given the same change in aromaticity, *ortho*‐quinones will possess a significantly more positive redox potential than non‐*ortho*‐quinones. By varying carbonyl placement in otherwise unfunctionalized quinones, the redox‐potential may be tuned by a range of up to 0.8 V. One previously uninvestigated quinone for ECCS, 2,3‐naphthoquinone, is identified as particularly promising, and nuclear magnetic resonance (NMR) spectroscopy shows its dianion capable of capturing CO_2_ but lacks electrochemical reversibility.

## Graphical abstract

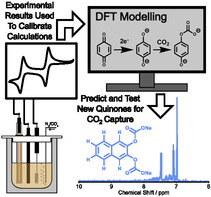

## 1 Introduction

It is now well‐known that the ever‐increasing level of CO_2_ present in our atmosphere is leading to unprecedented climate change in the history of our planet. The Intergovernmental Panel on Climate Change have reported with high confidence that the mean global surface temperature has risen by 1.09 °C since the period 1850–1900 and that this temperature rise is almost entirely attributable to human‐driven activities increasing the concentration of well‐mixed greenhouse gases in the atmosphere.^[^ ^1^ ^]^ One technology that can assist in mitigating these emissions is CO_2_ capture, where CO_2_ is either removed from a point source, such as factory flue gas, or removed directly from the air.^[^ ^2^, ^3^ ^]^

Amine‐scrubbing is a mature technique of industrial‐scale CO_2_ capture which has been developed for the last 90 years.^[^ ^4^ ^]^ In this case, the mechanism of capture is thermal‐swing, where the amines are regenerated and the CO_2_ released via stripping with steam.^[^ ^5^ ^]^ However, this is an energy intensive method, with commercial plants reporting an energy cost of 107 kJ per mole of CO_2_ captured from a flue gas containing 10.3% CO_2_.^[^ ^6^ ^]^ This is significantly higher than the thermodynamic limit of isothermally concentrating an ideal gas from 0.103 to 1 atm, which is calculated to be 5.6 kJ mol^−1^ at 298.15 K (Section S1, Supporting Information).

Electrochemical CO_2_ capture and storage (ECCS) offers an alternative to thermal‐swing based mechanisms with the possibility of accessing much greater energy efficiency.^[^ ^7^ ^]^ A variety of ECCS systems have been shown to be promising, such as pH‐swing,^[^ ^7^, ^8^, ^9^ ^]^ where energy efficiencies of 36 kJ mol^−1^ have been reported^[^ ^9^ ^]^ under an atmosphere of 10% CO_2_ and 90% N_2_. The use of redox‐active capture molecules in a nucleophilic‐swing mechanism has also been shown to be a successful ECCS method,^[^ ^7^, ^10^, ^11^, ^12^, ^13^ ^]^ with the most extensively studied class of redox‐active molecules for ECCS being quinones.

Quinones are a class of molecule which are abundant in nature, where their associated redox reactions affect a wide range of biological processes.^[^ ^14^ ^]^ Outside of biology their electrochemistry has been extensively studied in both aprotic^[^ ^15^ ^]^ and aqueous^[^ ^16^ ^]^ solvent systems. It was first shown that quinones can be used as a redox‐active sorbent for ECCS in aprotic organic solvents,^[^ ^17^, ^18^ ^]^ but more recently it has been shown that ECCS can also be achieved in aqueous systems.^[^ ^19^ ^]^ Unfortunately both organic and aqueous quinone‐based systems suffer from sensitivity to oxygen.^[^ ^17^, ^19^ ^]^

For the majority of quinones studied for ECCS, the potential at which they are reduced to their dianion state (the state which captures the CO_2_) is negative of the potential at which molecular oxygen is reduced to superoxide $\left(O_{2}^{•-}\right)$.^[^ ^20^, ^21^, ^22^ ^]^ This reduction of O_2_ is a parasitic reaction and therefore whenever oxygen is present, the Coulombic efficiency of the ECCS system is lowered.^[^ ^20^ ^]^

Unfortunately, it has been noted multiple times in the literature that there is a trade‐off between the quinone redox potential and its ability to capture CO_2_.^[^ ^10^, ^21^ ^]^ Many quinones with relatively positive redox potentials have been synthesized, however, the most often found case is that quinones which may be reduced at a potential positive of the O_2_/$O_{2}^{•-}$ potential, do not capture CO_2_ strongly enough for practical use in ECCS.^[^ ^21^ ^]^

In this work, density functional theory (DFT) is first used to predict the trade‐off between redox‐potential and strength of CO_2_ binding for various common quinone frameworks, with the accuracy verified through cyclic voltammetry results. A Hückel model is then devised to demonstrate how the redox potential depends on both the change in aromatic stabilization after two‐electron reduction, as well as the relative carbonyl positions. Finally, it is predicted that 2,3‐naphthoquinone may be a promising quinone framework for further functionalization due to its predicted favorable redox potential and CO_2_ binding strength.

Overall this work demonstrates that the carbonyl positioning in quinones strongly influences the position of the trade‐off between redox ability potential and ability to capture CO_2_. Therefore, this works suggests that in the effort to overcome the O_2_ sensitivity of quinones, careful choice of the underlying structure is vitally important, and that further functionalization should in fact be a secondary consideration.

## 2 Results and Discussion

### 2.1 Reaction Scheme Considered

In polar aprotic solvents, quinones generally undergo two redox events at potentials within the solvent‐window, corresponding to the ${\text{Q/Q}}^{•-}$ and $Q^{•-}{\text{/Q}}^{2-}$ redox couples,^[^ ^15^ ^]^ Equation (1) and (2), respectively.

(1) $$Q+e^{-}\to Q^{•-}$$

(2) $$Q^{•-}+e^{-}\to Q^{2-}$$

It was found by Bui et al. that the radical semiquinone, $Q^{•-}$, electronic structure is significantly more challenging to accurately model than the quinone, or quinone dianion, which introduced errors when trying to predict the two redox couples separately.^[^ ^10^ ^]^ To eliminate the need for an accurate radical electronic structure, the two‐electron redox potential can instead be considered, Equation (3).

(3) $$Q+{\text{2e}}^{-}\to Q^{2-}$$

**FIGURE 1:**
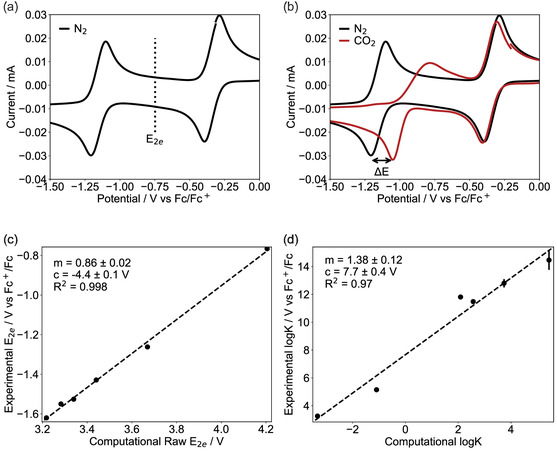
a) Cyclic voltammogram (CV) for terachloro‐benzoquinone (BQ‐Cl_4_) in DMSO with scan rate of 20 mVs^−1^ after purging solution with N_2_. Two one‐electron redox waves are present at −0.341 and −1.155 V, respectively. The experimental two‐electron redox potential (E_2*e*_), which is the average of the two one‐electron redox potentials, is also shown at −0.748 V. b) CVs for BQ‐Cl_4_ in DMSO after purging with N_2_ (black) and CO_2_ (red). Δ*E* is the positive shift in the more negative redox potential after purging with CO_2_, it is measured using the shift in the reduction wave. c) Comparison of raw E_2*e*_, calculated from computational methods (Section S2, Supporting Information) and the experimental E_2*e*_ under N_2_ referenced against the Fc^+^ /Fc potential. A line of best fit determined through linear regression analysis is also plotted, and the gradient (m), y‐intercept (c), and *R* ^2^ values for this line are given. d) Comparison of computational log(*K*), corresponding to capture of one‐equivalent CO_2_, to experimental values calculated using analysis of reduction peak shift observed in CV at varying CO_2_ concentrations. Again a line of best fit is determined and the associated parameters included in the figure.

The two‐electron redox potential, E_2*e*_, is the average of the two one‐electron redox potentials for the quinone. This can be determined experimentally using cyclic voltammetry, **Figure** **1**a, under a nitrogen atmosphere.^[^ ^10^ ^]^

**FIGURE 2:**
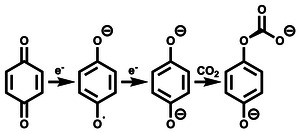
Example of “EEC” mechanism for benzoquinone. The first two steps are one‐electron reductions of the quinone, “E”. The final step is capture of CO_2_ via formation of a carbonate group, “C”.

There is debate as to whether the mechanism of CO_2_ capture in polar‐aprotic solvents is “ECE”, “ECEC”, “EEC”, or “EECC”, where “E” and “C” correspond to electrochemical and CO_2_ capture steps, respectively.^[^ ^7^ ^]^ Both “EEC”^[^ ^23^ ^]^ and “ECEC”^[^ ^18^, ^22^ ^]^ have been reported for different quinones, but whenever the binding constant to the semiquinone has been calculated, it has been found to be significantly smaller than the dianion binding constant.^[^ ^7^ ^]^ In this work, an “EEC” mechanism is implicitly assumed (**Figure** **2**), avoiding the requirement of an accurate treatment of the radical species.
(4)

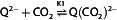




In this work then, *K*_1_ (Equation (4)), refers to the equilibrium constant corresponding to capture of exactly one equivalent of CO_2_ by the quinone dianion. Note however that the standard experimental method of calculating the CO_2_ binding constant, *K*_n_, is based on a different equilibrium equation (Equation (5)).^[^ ^7^, ^20^, ^23^ ^]^

(5) $$Q^{2-}+{\text{nCO}}_{2}\;\overset{\text{Kn}}{⇌}\;Q{\left({\text{CO}}_{2}\right)}_{n}^{2-}$$

The value of ln *K*_n_ is experimentally determined from cyclic voltammetry, using the shift in the $Q^{•-}{\text{/Q}}^{2-}$ redox potential, Δ*E*, at various CO_2_ concentrations from its value under 100% N_2_, normally approximated by the shift in the corresponding reduction wave, Figure 1b. This value of *K*_n_ is extracted using Equation (6), where *F,R,T*, and [CO_2_] correspond to the Faraday constant, the gas constant, the temperature, and the CO_2_ concentration, respectively. For a detailed derivation see Section S3.4, Supporting Information.

(6) $$\ln K_{n}=\frac{F}{RT}\Delta E-n\ln[{\text{CO}}_{2}]$$

This equilibrium equation is not used for the computational calculations as the noninteger possible values of *n* (*n* can be thought of as the average number of CO_2_ captured per quinone) mean that the equilibrium constants, *K*_n_, are not technically comparable.

In this work, however, equilibrium constants calculated for single‐capture, *K*_1_, are calibrated against the general *n*‐capture equilibrium constants, *K*_n_, obtained from cyclic voltammetry measurements. From here on both are referred to simply as *K*. Ideally, results would be bench‐marked against experimental values of single‐capture equilibrium constants, however, there is not yet reported a standard way of extracting these values from the cyclic voltammetry results in this type of system.

### 2.2 Comparison and Calibration of Computational and Experimental Results

The two‐electron redox potentials and CO_2_ binding constants for various quinones in DMSO are calculated from cyclic voltammetry (Section S3, Supporting Information). For the more strongly binding quinones, extra analysis was required to extract the binding constants and this is thoroughly discussed in Section S3.8 and S3.9, Supporting Information. For two of the quinones considered (1,4‐difluoroanthraquinone and 1‐chloroanthraquinone), the mechanism of CO_2_ capture had to be more carefully considered, with a full analysis given in Section S3.10, Supporting Information. Through linear regression analysis, these experimental results were used to assess the validity of the computational methods (described in Section S2, Supporting Information) and to provide a calibration, see Figure 1c,d as well as Section S4, Supporting Information.

When calibrating both E_2*e*_ and log(*K*), a large value of R^2^ is obtained, indicating a strong correlation between the results obtained from DFT and those calculated from cyclic voltammetry. From this found correlation, it can be assumed that the DFT results obtained later in this work should translate reasonably well to experimental values, and that this translation can be accomplished using the linear regression analysis values obtained in Figure 1c,d.

### 2.3 Trade‐off Between E_2*e*_ and Log(*K*)

**FIGURE 3:**
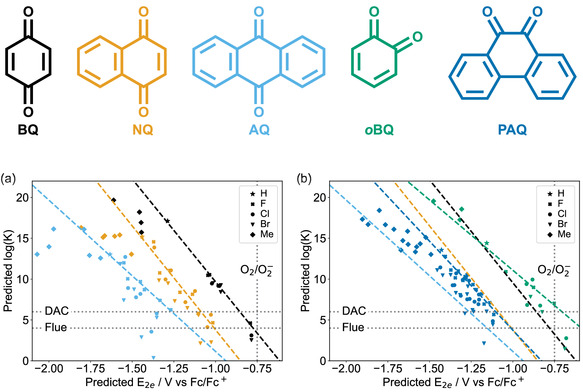
(Top Legend) The five quinone base structures investigated in the first part of this study, in order from left to right: benzoquinone (BQ), naphthoquinone (NQ), anthraquinone (AQ), *ortho*‐benzoquinone (*o*BQ), and phenanthrenequinone (PAQ), respectively. a,b) The five quinones shown in the top of the figure are symmetrically functionalized with a variety of functional groups (F, Cl, Br, Me). The computational methods described in Section S2, Supporting Information, are used to calculate log(*K*) and E_2*e*_, with the calibrations from Figure 1c,d used to convert these values to predicted experimental values. The predicted log(*K*) values are plotted against the predicted E_2*e*_ values to demonstrate the trade‐off between the two parameters. The unfunctionalized quinones are also considered and indicated with a star in the two figures. The vertical line at −0.75 V, labeled $O_{2}{\text{/O}}_{2}^{-}$, corresponds to the E_2*e*_ at which it is expected only 1% of O_2_ would be reduced to $O_{2}^{•-}$. The horizontal line at log(*K*) = 4 represents the minimum log(*K*) for applicability in CO_2_ capture from flue gas and the horizontal line at log(*K*) = 6 represents the minimum log(*K*) required for DAC. The lines of best fit are for only the F‐substituted quinones as these give the largest values of log(*K*) and thus are representative of the “best” trade‐off for the specified quinone. The *para*‐quinones (BQ, NQ, AQ) trade‐offs are shown in (a), and the *ortho*‐quinones (*o*BQ, PAQ) trade‐offs are shown in (b), with the lines of best fit from the *para*‐quinone plot included for comparison.

Although the number of possible quinone base structures (the delocalized carbon framework plus carbonyl position) is practically limitless, in practice only a few are generally considered. The most commonly studied quinone types are benzoquinone (BQ), naphthaquinone (NQ), anthraquinone (AQ), *ortho*‐benzoquinone (*o*BQ), and phenanthrenequinone (PAQ), **Figure** **3**.^[^ ^7^, ^23^, ^24^ ^]^ These quinones and many of their derivatives are readily available, with many possessing reversible electrochemistry.

In this work, the redox potentials and CO_2_ binding constants for the derivatives of these five quinones are explored using computational methods. In each case, the quinones were symmetrically substituted with one type of halide or with methyl groups (chosen to minimize effects of steric‐hindrance or other intramolecular effects such as H‐bonding) so that both oxygens remained equivalent. This ensured that for each quinone only one value of log(*K*) had to be considered. The two‐electron redox potentials and single‐capture equilibrium constants were then calculated and compared with each other (Figure 3a,b and Tables S5–S9, Supporting Information).

For naphthoquinones, the asymmetric fluorinated derivatives were also studied and it was found they followed the same trends as the symmetric derivatives (Section S8, Supporting Information). Therefore for the other quinones, we assume similarly that the trends derived from the symmetric derivatives are representative of the wider space of possible species.

For each of the five types of quinones in Figure 3, we find that they possess unique linear free‐energy relationships, which is not unexpected, and agrees with previously reported results, including the observation that benzoquinones possess a more favorable trade‐off.^[^ ^10^, ^23^ ^]^ It was observed that out of the substitutions investigated, F‐substitutions gave the most promising trade‐off line (highest log(*K*) at a given E_2*e*_.) Because of this, the presented lines‐of‐best fit in Figure 3a,b are those corresponding to the trade‐offs for substituting the quinones with fluorine. These lines of best fit then represent the best‐case trade‐off line when only looking at F, Cl, Br, and methyl groups.

Out of the five types of quinone investigated here, *ortho*‐benzoquinone was the sole type with a trade‐off line intercepting the direct air capture (DAC)‐possible region, Figure 3b. For a discussion on how the potential required to avoid O_2_ reduction was determined, please refer to Section S7, Supporting Information. The trade‐off line for benzoquinone is the next most promising, very close to intercepting the flue‐gas possible region Figure 3a. The remaining three types (NQ, AQ, and PAQ) possess trade‐off lines much further from either the DAC‐possible or flue‐gas possible regions. Despite the interception of the DAC‐region, *ortho*‐benzoquinone itself (green star, Figure 3b) is quite removed from the DAC‐possible region, and requires significant functionalization to be brought in to it, but only possesses four possible sites which can be functionalized. There are also issues of stability with *ortho*‐benzoquinone, and even relatively stable types may not possess reversible electrochemistry (Figure S14, Supporting Information).

To summarize these first results: 1) each of the five quinone types investigated in this section possess a unique linear free‐energy relationship relating log(*K*) to E_2*e*_; 2) for each of the five quinone types, the most promising trade‐off (highest log(*K*) at a given E_2*e*_) is attributed to the fluorine substitutions; 3) none of the quinones investigated here are predicted to be suitable for application in CO_2_ capture from either flue gas or directly from the air; 4) *ortho*‐benzoquinone is the only quinone studied where the trade‐off line intercepts the DAC‐possible region.

### 2.4 Predicting Aromatic Stabilization upon Reduction

From the last section, two results stood out significantly: the quinone type has a large impact on whether the trade‐off between log(*K*) and E_2*e*_ intercepts the DAC‐possible region and that the unfunctionalized quinones all possess a relatively negative E_2*e*_. The aim then, was to find a new type of quinone with a trade‐off line which intercepts the DAC‐possible region, and which ideally would already be in, or close to, the DAC‐possible region, so that a minimal amount of functionalization would be required. To achieve this goal, we first investigated how the structure of a quinone influences the predicted two‐electron redox potential.

We theorized that the major difference between the different quinone types, was the aromatic stabilization upon two‐electron reduction (i.e., the change in energy of the delocalized *π*‐system). To investigate this hypothesis, we devised a Hückel model which could be applied to the neutral and dianion quinone states. For this model, some key assumptions were made: 1) For the neutral quinone, the carbonyl bonds are localized, with a defined energy, and do not participate in the delocalized ring *π*‐system of the ring. The energy of the carbonyl bonds are equal for all quinones; 2) After two‐electron reduction, the carbonyl bonds are broken, and the former carbonyl‐carbon is allowed to conjugate with the *π*‐system. The oxygen atoms do not interact with the ring *π*‐system; 3) The resonance interaction between neighboring, conjugating carbons is equal for all quinones and is equal in both the neutral and dianionstates.

#### 2.4.1 Example Aromatic Stabilization Calculation Using Benzoquinone

To illustrate how the model derived from the assumptions made above can be utilized to extract a quantitative value of aromatic stabilization upon two‐electron reduction, we demonstrate here how the calculation is performed for benzoquinone.

##### 2.4.1.1 Neutral Quinone

**FIGURE 4:**
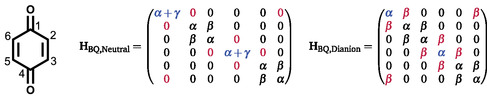
Left: Neutral benzoquinone, with numbered carbon atoms. Middle: Constructed Hückel matrix for neutral benzoquinone, assuming that the carbonyl bonds are localized with energy *α* + *γ* and that the resonance interaction between neighboring noncarbonyl carbons (in this case 2, 3, 5, and 6) is *β*. Right: Constructed Hückel matrix for the benzoquinone dianion, assuming that there are now resonance interactions between all neighboring carbons and that the electron density on each carbon is equal.

It is assumed that the carbonyl *π*‐bonds are localized with energy *α* + *γ*. It is then assumed that the remaining carbon atoms each possess one available *p*‐orbital for *π* bonding, all with energy *α*. The last assumption is that the only resonance interactions are between neighboring, noncarbonyl carbons, with a strength of *β* (*β* < 0). These assumptions are utilized to construct the appropriate Hückel matrix, **H**_BQ,Neutral_ (**Figure** **4** middle).

##### 2.4.1.2 Dianion Quinone

It is now assumed that there is no *π* interaction with the oxygen atoms and that every carbon possesses a *p*‐orbital available for *π* bonding, with energy *α*. It is also assumed that there are now resonance interactions (strength *β*) between all neighboring pairs. These assumptions are again utilized to construct an appropriate Hückel matrix, **H**_BQ,Dianion_ (Figure 4 right).

The orbital energies for the neutral and dianion states are found by solving the matrix equation (Equation (7)). In this case **H** is the Hückel matrix being used, **C** is the orbital coefficients matrix, where each column vector corresponds to a normalized *π* orbital of the system. **E** is a diagonal matrix with the diagonal values corresponding to the orbital energies of the *π*‐system.

(7) HC=EC

**FIGURE 5:**
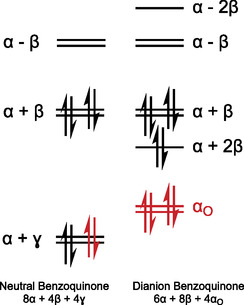
The *π*‐MO diagram for neutral benzoquinone (left) and the benzoquinone dianion (right) according to the described Huckel model. Electrons originating from the oxygen atoms are colored red. The total calculated energies of the two *π* systems are provided below the respective MO diagrams.

The energy levels are then found (including assumed energy of localized oxygen lone pairs) and correctly populated (from lowest to highest energy) to obtain a total energy for the *π*‐system in both the neutral and dianion states (**Figure** **5**).

For every quinone considered, following the above model will always result in the neutral energy having a contribution of 2*α* + 4*γ* from the carbonyl bonds, and the dianion energy will have an energy contribution of 4*α*_*o*_ from the oxygen lone pairs. These terms are assumed constant for all quinones with the remaining difference in energies given only in terms of *β*, this can therefore be compared between different quinones. This is the value we consider to be the change in aromatic stabilization upon two‐electron reduction, *χ*, Equation (8).

(8) $$\chi =E_{\text{Dianion}}-E_{\text{Neutral}}-4\alpha_{O}+2\alpha +4\gamma$$

Using the MO diagrams in Figure 5 and Equation (8), it is calculated that for benzoquinone, χ=4β.

We note that the MO diagrams generated from the Hückel calculations (Figure 5) should not be expected to match those obtained via DFT and are likely significantly different. The authors wish to be clear that this Hückel model is not designed to provide accurate energies of quinones, but instead provide semi‐quantitative insight into the role of aromaticity confined to the carbon frameworks.

#### 2.4.2 *Calculating* *χ* *for a Range of Quinones*

**FIGURE 6:**
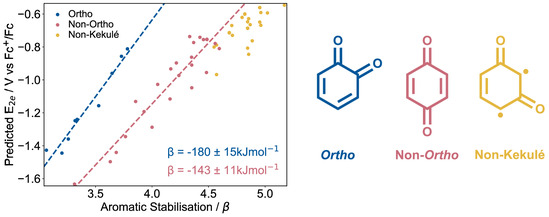
Left: Predicted E_2*e*_ values for all possible unsubstituted benzoquinones, naphthoquinones, anthraquinones, and phenanthrenequinones, plotted against the calculated aromatic stabilization from the outlined Hückel theory. Linear regression is performed on the *ortho* and non‐*ortho* quinone data sets separately and lines of best fit plotted accordingly. Values of *β* are derived from the line of best fit gradients (Section S9, Supporting Information). Right: Examples of an *ortho*, non‐*ortho*, and non‐Kekulé quinone structure. Non‐Kekulé quinones are those where the delocalized *π*‐system cannot be represented without including radicals.

To investigate how the carbonyl positioning in a quinone affects the aromatic stabilization upon reduction, and the predicted value of E_2*e*_, we used benzene, naphthacene, anthracene, and phenanthrecene as the starting carbon‐frameworks. For each of these structures, all possible carbonyl placements were considered and the values of *χ* were calculated using the methodology from the previous subsection (Table S13, Supporting Information). The values of E_2*e*_ were also calculated, using the same computational methods (Section S2, Supporting Information) and calibrations described earlier (Figure 1c,d). The set of generated quinones were partitioned into those which are *ortho* (where the carbonyls are *ortho* to each other), non‐Kekulé (where the carbonyl placement makes it impossible to draw the quinone structure without radicals), and the remaining quinones were allocated the non‐*ortho* group (**Figure** **6** right).

Excitingly a strong correlation is observed between E_2*e*_ and *χ*, as hypothesized, especially for the *ortho* quinones (Figure 6). This result demonstrates that there is a strong relationship between the aromatic stabilization and E_2*e*_ for the quinones investigated here.

The primary finding from these results is that by varying the carbonyl placement in the quinone, E_2*e*_ can be tuned by ≈0.8 V. This is comparable to the degree of tuning one can obtain by changing the functional groups on a single type of quinone.

Interestingly, it was found that the relationship between *χ* and E_2*e*_ is significantly different between the *ortho* and non‐*ortho* sets. At the same quantity of aromatic stabilization upon reduction, the E_2*e*_ for *ortho* quinones are more positive than non‐*ortho* quinones, that is, they are easier to reduce. We then hypothesize that if strength of CO_2_ capture is dependent on *χ*, that *ortho*‐quinones should on average possess larger values of log(*K*) at more positive redox potentials, and are therefore potentially promising candidates for ECCS.

We also note that all of the non‐Kekulé quinones are predicted to have both very large *χ* and also relatively positive values of log(*K*). We do not further consider these molecules for application in CO_2_ capture however as synthesizing these molecules would likely be challenging and their radical nature would likely make these molecules relatively unstable.

### 2.5 Evaluating the Unsubstituted Quinones’ Capability for CO_2_ Capture

We have shown that by varying the quinone carbonyl position on five common carbon‐frameworks, the two electron redox potential can be tuned over a range of ≈0.8 V. It is also predicted that at a given quantity of predicted aromatic stabilization upon two‐electron reduction, *ortho* quinones are predicted to possess a more positive E_2*e*_ and therefore potentially be promising candidates for ECCS. To test this hypothesis, we predicted log(*K*) for each of the unsubstituted quinones and compared the results to the trade‐off lines obtained via substitution of the common quinones in Figure 3. For these calculations note that for the majority of new quinone structures generated, the two oxygen sites are not symmetrically equivalent, in which case two values of log(*K*) were calculated, corresponding to capture of CO_2_ at the two nonequivalent sites respectively.

**FIGURE 7:**
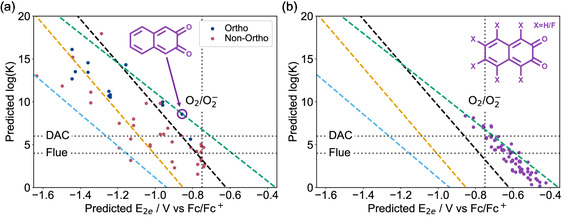
a) The predicted log (*K*) values for the unsubstituted quinones which were investigated in the previous section, plotted against the predicted E_2*e*_ values. Also shown are the fluorine‐substituted trade‐off lines for benzoquinone (black), *ortho*‐benzoquinone (green), naphthoquinone (yellow), and anthraquinone (light blue). The data point corresponding to 2,3‐naphthoquinone is circled and its structure given (purple). b) The predicted log(*K*) values plotted against the predicted E_2*e*_ values for all the possible fluorine‐substituted 2,3‐naphthoquinones. When the functionalization led to asymmetric oxygen sites, log(*K*) was calculated at both oxygen sites and both data points included in the figure.

Interestingly, but disappointingly, the results in **Figure** **7**a indicate that the previously identified trade‐off lines from Figure 3 act as bounds for this new data set, with almost all the unsubstituted quinones predicted to lie between the *ortho*‐benzoquinone and anthraquinone trade‐off lines. This possibly indicates that to break out of these bounds, alternative carbon‐frameworks for quinones should be investigated.

In regards to the difference in CO_2_ capture performance between *ortho* and non‐*ortho* quinones, there appears to be a slight preference for *ortho* quinones to have higher values of log(*K*) at a given E_2*e*_, however, the preference is only small (Figure 7a). When log(*K*) is compared to aromatic stabilization, it is found that the correlation is quite weak and approximately equal for both *ortho* and non‐*ortho* quinones (Figure S21, Supporting Information).

Out of the quinones evaluated in Figure 7a, although none were found to significantly lie above the *ortho*‐benzoquinone trade‐off line, one promising candidate was identified, 2,3‐napthoquinone (see Lewis structure in Figure 7b). This quinone was predicted to lie directly on the *ortho*‐benzoquinone line, but has the advantage that it is predicted to lie significantly closer to the DAC‐possible region than *ortho*‐benzoquinone, and has six possible sites for functionalization as opposed to *ortho*‐benzoquinone's four, providing ample sites for further tuning E_2*e*_ and log(*K*).

### 2.6 Trade‐off for Fluorine Substituted 2,3‐Naphthoquinone

To further investigate 2,3‐naphthoquinone, the two‐electron redox potentials and CO_2_ binding constants were calculated for all the possible fluorine‐substituted derivatives, Figure 7b. Extremely promisingly, three of the derivatives were predicted to lie in the DAC‐possible region, and a further fourteen were predicted to lie in the flue‐gas region. This is a significant improvement on the results of the halide and methyl functionalized quinones investigated earlier, Figure 3, where no quinone was predicted to even be suitable for CO_2_ removal under flue gas conditions.

On another interesting note, the trade‐off fairly well follows the previously identified trade‐off for *ortho*‐benzoquinone, Figure 7b. This is perhaps unsurprising as one way to think of 2,3‐naphthoquinone is as a *ortho*‐benzoquinone functionalized with an additional conjugated ring.

In summary, these very promising results indicate that tuning the underlying quinone structure, and then functionalization, may be the optimal method for discovering new quinones for application in ECCS. Additionally, being able to design quinones with even more positive redox potentials than previously seen should be of particular interest in the development of cathodes in organic redox‐flow batteries.

### 2.7 Experimental Results for 2,3‐Naphthoquinone

After promising computational predictions for 2,3‐naphthoquinone and its derivatives, an attempt was made to experimentally verify said predictions. It was found that the hydroquinone is commercially available, thus oxidation to the quinone was attempted, but NMR analysis showed that the obtained product was impure and had possibly undergone polymerization (Section S10.2, Supporting Information). The oxidized quinone being unstable would be unfortunate but not inexplicable as *ortho*‐benzoquinone is also known to be unstable.^[^ ^25^ ^]^

**FIGURE 8:**
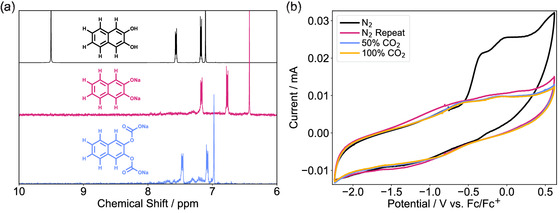
a) ^1^H solution‐state NMR Spectra in D_6_‐DMSO on a 400 MHz machine of 2,3‐dihydroxynapthalene (black), the sodium salt of the 2,3‐naphthoquinone dianion (red), and the same dianion after bubbling with CO_2_ (blue). Only the ppm range of interest is shown, full spectra and peak assignments presented in the Figure S25, Supporting Information. All spectra display a singlet, and two doublets of doublets in the shown region, the hydroquinone also possesses a singlet at 9.5 ppm corresponding to the —OH group. b) 100 mVs^−1^ CVs of the 2,3‐naphthoquinone dianion sodium salt (5 mM) in DMSO with TBAPF_6_ (0.1 M) supporting electrolyte. For each separate CV experiment, three cycles were recorded, the last of which is shown in this figure. There was a gap of approximately two hours between the two CV experiments under N_2_.

Despite not isolating the oxidized quinone, isolation of the quinone dianion was sought via deprotonation of the hydroquinone (Section S10.3, Supporting Information), which proved more successful. The ^1^H NMR spectrum of the dianion was obtained and after bubbling the sample solution with CO_2_, the ^1^H NMR spectrum then indicated capture of CO_2_ via the dianion, **Figure** **8**a. Interestingly, the retention of symmetry in the NMR spectrum after bubbling with CO_2_ indicated that the quinone dianion is capturing one molecule of CO_2_ at each oxygen site, or that a single molecule of captured CO_2_ switches between the two oxygen sites faster than the NMR timescale, however, we believe that the first of these two options is the more likely.

After demonstrating that the dianion of 2,3‐naphthoquinone is capable of CO_2_ capture, we attempted to measure the strength of this binding using cyclic voltammetry (Figure 8b). The first CV under N_2_ appeared exciting for two reasons, the open‐circuit potential was remarkably positive, at ≈−0.75 V versus Fc^+^/Fc, which was significantly positive of the predicted E_2*e*_. Then while scanning in the positive direction, two low intensity and poorly resolved oxidation peaks were observed. Unfortunately no corresponding reduction peaks were seen, indicating that any oxidation to the quinone itself is irreversible. Also note that when the CV was repeated after leaving the solution for two hours under N_2_, the oxidation peaks were no longer present and the sample was effectively electrochemically inactive (Figure 8b).

When the sample was purged with various concentrations of CO_2_, the sample appeared to remain electrochemically inactive, however, the same color changes occurred as had been observed when purging the NMR sample with CO_2_, possibly indicating that the bulk electrochemical solution had not degraded, but that the issue may lie at the working electrode. When ferrocene was added at the end to take an internal reference against, the ferrocene/ferrocenium signal was unusually low intensity and ill‐defined, again indicating that the issue lay at the working electrode. Upon cleaning the setup, it was noted that there appeared to be a greasy film on the working electrode, which would explain the loss of oxidation waves under N_2_ and also the poorly defined ferrocene signal. We believe that this, along with the result of attempting to chemically synthesize the oxidized quinone, indicate that 2,3‐naphthoquinone is inherently unstable, and therefore experimental values of E_2*e*_ and log(*K*) could not be obtained via CV methods.

To further explore this computationally promising ECCS candidate, we aimed to improve the stability of the quinone such that reversible electrochemistry could be measured. We were able to chlorinate the hydroquinone at the two sites *ortho* to the hydroxy groups (Section S11.1, Supporting Information). Oxidation was tested under the same conditions used previously, where we do not believe that polymerization occurred, however, a reaction with water, required for the oxidizing agent, may have taken place, such that the expected quinone was not isolated (Section S11.5, Supporting Information). This result although not ideal points towards further functionalization possibly resulting in stability of the oxidized quinone.

After deprotonation of the chlorinated hydroquinone and exposure to CO_2_, the ^1^H NMR again indicated that the maximum of two equivalents of CO_2_ are captured per quinone dianion (Section S11.2 and S11.3, Supporting Information). Unfortunately, both the oxidized product and the sodium‐dianion salt showed no reversible electrochemistry (Section S11.6, Supporting Information), further discussion is given in Section S11.7, Supporting Information.

## 3 Conclusions

First, it was shown that computational predictions for two‐electron redox potentials, and CO_2_ binding constants, both correlate well to experimental values derived from cyclic voltammetry. From these correlations, we were able to calibrate our computational methods and further investigate how functionalization of the five most common quinones for ECCS affects their ability to capture CO_2_ and also their tolerance towards the presence of O_2_.

Out of the functionalized common quinones investigated, none were predicted to be capable of capturing CO_2_ strongly enough for use in DAC, while also being tolerant to the presence of O_2_. It was further shown that each of the five quinones possess a distinct trade‐off between redox potential and CO_2_ binding constant, which indicated that out of the five quinones, only *ortho*‐benzoquinone could potentially be further functionalized to be suitable for DAC.

The finding of distinct trade‐offs for the different quinones prompted us to further investigate the relationship between quinone structure and redox‐potential. Using Hückel theory, we found a strong correlation between change in aromaticity upon reduction, and two‐electron redox potential for unfunctionalized quinones. We further found that *ortho* quinones possess a more positive redox potential at the same calculated change in aromaticity upon reduction.

When the CO_2_ binding constants were calculated for the unfunctionalized quinones, it was found that the majority were bound by the previously identified trade‐off lines for the common quinones. Although no quinones were identified, which seemed to outperform the *ortho*‐benzoquinone trade‐off line, 2,3‐naphthoquinone was identified as a promising candidate due to lying on the *ortho*‐benzoquinone trade‐off line, proximity to the O_2_‐tolerant DAC region, and six possible sites for further functionalization.

When the effect of fluorine functionalization on 2,3‐napthoquinone was calculated, there were three derivatives predicted to lie in the O_2_‐tolerant DAC region and an additional fourteen were predicted to be potentially suitable for CO_2_ removal from flue gas.

Finally, we attempted to obtain experimental verification of the redox potentials and CO_2_ binding constant for 2,3‐naphthoquinone. While it was shown using NMR spectroscopy that the dianion is capable of capturing two‐equivalents of CO_2_, the oxidized form of the quinone is unstable and the two‐electron redox potential and CO_2_ binding constant could not be extracted fromcyclic voltammetry. When the experiment was repeated for a chlorinated derivative of the quinone, stability of the oxidized quinone possibly improved, however reversible electrochemistry was still not observed. The authors are optimistic however that if stable derivatives of this quinone can be synthesized, that they will be of considerable interest to the ECCS community.

Overall this study finds that despite different quinones possessing distinct trade‐off lines, there are none which significantly pass through the DAC‐possible region. However, some quinones have considerably more promising trade‐offs than others, and therefore, this article shows that selecting an appropriate underlying structure is vital for achieving O_2_ resistance with sufficiently strong binding to CO_2_. Once an underlying structure is chosen then further tuning may be accomplished via functionalization, and further tuned into the DAC‐possible region by the addition of extra effects not considered in this article such as intramolecular interactions^[^ ^13^ ^]^ or through the use of additives in the electrolyte.^[^ ^12^ ^]^ We hope that the results of this article will help guide the development of the next‐generation of redox‐active sorbents for CO_2_ capture.

## Conflict of Interest

The authors declare no conflict of interest.

## Supporting information

Supplementary Material

## Data Availability

QChem output files and cyclic voltammetry data are available at https://doi.org/10.17863/CAM.117774.
