# Supplementary material for: Impacts of Quinone Structure on Trade‐Offs Between Redox Potential and CO2 Binding Strength
Source: Chemphyschem. 2026 Apr 7;27(7):e202500472. doi: 10.1002/cphc.202500472 (PMC13056350; doi:10.1002/cphc.202500472)
Supplement: Supplementary file 1 — Supplementary Material [file CPHC-27-e202500472-s001.pdf]

Impacts of Quinone Structure on Trade-Off Between Redox Potential and CO_2 Binding Strength

Supplementary Information

Jack.S.Taylor, Alexander.C.Forse, Alex.J.W.Thom

October 24, 2025

Contents

S1 Concentrating an Ideal Gas **4**

S2 Computational Methods **5**

S2.1 Calculating Two-Electron Redox Potentials and CO_2 Binding Constants 5

S2.2 Geometry Optimisation 5

S3 Experimental Determination of CO_2 Binding Constants and Two-Electron Redox Potentials **6**

S3.1 Cyclic Voltammetry Experimental 6

S3.2 CV Results at 20 mVs^{-1} 7

S3.3 CV Results at 100 mVs^{-1} 8

S3.4 Derivation of log$K$ from Shift in Second Reduction Wave at Variable CO_2 Concentrations 9

S3.5 Shift vs CO_2 Concentration Plots at 20 mVs^{-1} 11

S3.6 Shift vs CO_2 Concentration Plots at 100 mVs^{-1} 12

S3.7 Summary of Experimental Values for log$K$ and E_{2e} 13

S3.7.1 20 mV s^{-1} 13

S3.7.2 100 mV s^{-1} 13

S3.8 Isolating Second Reduction Peak 14

S3.8.1 Method 14

S3.8.2 Discussion on CV Results 15

S3.8.3 Plots and Discussion of New Values for n and log(K) 15

S3.8.4 Tabulated Corrected Experimental Values for log$K$ and E_{2e} 16

S3.9 Validation of Reference Subtraction Method 17

S3.9.1 CV Simulation Methodology 17

S3.9.2 Results 17

S3.10 Further Consideration of Double Peak Behaviour 19

S3.10.1 Tabulated Fully Corrected Experimental Values for log$K$ and E_{2e} 23

S4 Calibration of Computational Results **24**

S4.1 Discussion of Calibration Results 24

S5 Tetrachloro-1,2-benzoquinone **25**

S6 Tabulated Results for Substituted Quinones **26**

S6.1 Benzoquinones 26

S6.2 Naphthoquinones 26

S6.3 Anthraquinones 27

S6.4 *ortho*-Benzoquinones 27

S6.5 Phenanthrenequinones 28

S7 E_{2e} to Avoid Oxygen Reduction **29**

S8 Considering Asymmetric Quinones **30**
S8.1 Tabulated Results for Asymmetrically Substituted Naphthoquinones 31

S9 Calculating Aromatic Stabilisation Energy **32**
S9.1 Calculating β 32
S9.2 Tabulated Results for Aromatic Stabilisation Study 33
S9.3 Relationship Between CO_2 Binding Constant and Aromatic Stabilisation 34

S10 2,3-Naphthoquinone **35**
S10.1 Tabulated Results for Fluorinated 2,3-NQ 35
S10.2 Attempt to Synthesise 2,3-Naphthoquinone 36
S10.3 Synthesis of 2,3-Naphthoquinone Dianion 37
S10.4 Electrochemistry 38

S11 Dichloro-2,3-Naphthoquinone **39**
S11.1 Chlorination of Napthalene-2,3-diol 39
S11.2 Deprotonation of 1,4-Dichloronapthalene-2,3-diol 40
S11.3 Reaction of Sodium Salt with CO_2 41
S11.4 Comparison of the Three Synthesised Reduced Species of Dichloro-2,3-Napthoquinone . 42
S11.5 Oxidation of the Dichlorinated Hydroquinone 43
S11.6 Electrochemistry of the 1,4-Dichlor-2,3-Naphthoquinone Dianion 44
S11.7 Discussion on the Stability of 2,3-Naphthoquinone 44

S1 Concentrating an Ideal Gas

A gas is considered ideal if it follows the ideal gas law, Eq. (S1), where p, V, n, R, T correspond to pressure, volume of gas, moles of gas, the ideal gas constant and the absolute temperature respectively.

$$pV = nRT \tag{S1}$$

The work required to isothermally and reversibly concentrate an ideal gas from pressure p_i to p_f is given by Eq. (S2), where w is the work.

$$w = -nRT \ln\left(\frac{p_i}{p_f}\right) \tag{S2}$$

For concentrating an ideal gas from 0.103 to 1 atm at 298.15 K, the work required per mole is evaluated as $w/n = -R \times 298.15 \times \ln(0.103/1) = 5.6$ kJ mol^{-1}.

For derivation and further information, please refer to *Physical Chemistry* by Atkins.[1]

S2 Computational Methods

S2.1 Calculating Two-Electron Redox Potentials and CO_2 Binding Constants

For each quinone two structures, corresponding to the neutral quinone, and the dianion with CO_2 attached at one oxygen site, were created in IQMol and their geometries initially optimised using the UFF force field. The neutral geometry was used as the guess geometry for both the neutral and dianion quinones.

The geometries of the quinone, dianion, and CO_2-attached dianion were optimised at the B3LYP[2,3,4,5]/6-311++G**[6,7,8,9,10,11] level using C-PCM[12] to model solvation in DMSO (dielectric = 46.68). A frequencies calculation was then ran on the optimised geometry at the same level of theory to obtain the vibrational zero-point energy, as well as enthalpy and entropy contributions to the free energy.

The functional and basis-set were chosen to match those used in a previous study also investigating the relationship between redox-potential and CO_2 binding constant for quinones in organic solvent..[13]

The gibbs energy for each molecule considered was calculated according to Eq. (S3), where U, E_0, H, T, S correspond to the SCF energy, the vibrational zero-point energy, enthalpy contributions, temperature, and entropy contributions respectively

$$G^{\circ} = U + E_0 + H - TS \tag{S3}$$

The two-electron redox potential for each quinone is then calculated according to Eq. (S4), where F is the Faraday constant.

$$E_{2e} = \frac{G^{\circ}(\text{dianion}) - G^{\circ}(\text{quinone})}{-2F} \tag{S4}$$

Note that this corresponds to calculating the potential of a half cell-reaction where the free-energy of the reducing electrons is ignored, hence these values have to be calibrated against experimental results which share a common reference potential.

Values of $\ln K$ for binding a single CO_2 molecule to the quinone dianion were calculated according to Eq. (S5) where $G^{\circ}(CO_2)$ corresponds to the free energy of CO_2 in DMSO, calculated using the same computational methods as for the quinones. R and T correspond to the gas constant and the temperature respectively.

$$\ln K = \frac{G^{\circ}(\text{Dianion-CO}_2) - G^{\circ}(\text{Dianion}) - G^{\circ}(\text{CO}_2)}{-RT} \tag{S5}$$

All calculations were ran using Q-Chem[14] and assuming a temperature of 298.15 K

S2.2 Geometry Optimisation

The minima obtained through the geometry optimisation were first analysed using the analytical Hessians which resulted in imaginary frequencies all less than 100 cm^{-1}, with the corresponding normal modes corresponding to attachment and rotation of the CO_2 group. Despite attempts to remove these frequencies via displacement along the normal mode and re-optimisation, the imaginary frequencies persisted.

To further investigate this matter, the frequency calculation was repeated with the same geometry however the Hessian was calculated using a finite differences method triggered by setting "IDERIV = 1" in QChem. In every case the finite differences calculation indicated no imaginary frequencies and that the geometry is in fact a true minimum.

S3 Experimental Determination of CO_2 Binding Constants and Two-Electron Redox Potentials

S3.1 Cyclic Voltammetry Experimental

The electrolyte solutions for the CV experiments consisted of anhydrous DMSO (25 mL), 0.1M tetrabutylammonium hexafluorophosphate $TBAPF_6$ and 5mM of the quinone being investigated.

The electrochemical solution was decanted in to a 100 mL glass beaker and a bulk-electrolysis teflon cell top (BASi) was added. A small cylindrical stirrer bar was also added. The beaker was placed in a wider beaker containing aluminium beads which were used to heat the electrolyte solution.

Three electrodes were fitted into the cell top. For the reference electrode, a leak-free Ag/AgCl reference electrode within a fritted glass chamber was used. The working electrode was glassy carbon and the counter electrode a coiled platinum wire. All electrodes were sourced from BASi.

A thermometer was lowered directly into the electrolyte solution. Gas mixtures were controlled using mass flow controllers (Aalborg DPC controllers 0-500 smL min^{-1}) to mix different rates of pure N_2 and CO_2 respectively. The gas was output through a thin needle which could be lowered directly in to the solution. The outer rim of the 100 mL beaker was parafilmed to minimise exposure to air.

For each solution, the temperature was set to reach 25°C. While the solution reached this steady temperature the mixture was moderately stirred and purged constantly with pure N_2 (50 smL min^{-1}). Once a steady temperature had been reached and at least 15 minutes had elapsed, the stirring was stopped. The gas flow needle was raised above the solution so that the headspace gas mixture would be maintained without disturbing the solution. The CV corresponding to 0% CO_2 was then immediately taken.

After the cyclic voltammetry was completed, the gas flow needle was lowered into the solution and stirring commenced again. The gas mixture was altered to contain 20% CO_2 (molar percentage) but the total flow rate of 50 smL min^{-1} was maintained. For 20% CO_2 this corresponded to flow rates of 10 and 40 smL min^{-1} for CO_2 and N_2 respectively. This gas mixture was purged in to the solution for 15 minutes and then as before the needle raised out of the solution but with gas flow maintained and the cyclic voltammograms taken. This procedure was repeated for CO_2 concentrations of 40, 60, 80, and 100%.

Once cyclic voltammograms had been recorded at the various CO_2 concentrations, a small amount of ferrocene was added to the solutions, and an additional CV taken. The measured ferrocene/ferrocenium redox potential was then used as the reference to report the previous CVs against.

S3.2 CV Results at 20 mVs^{-1}

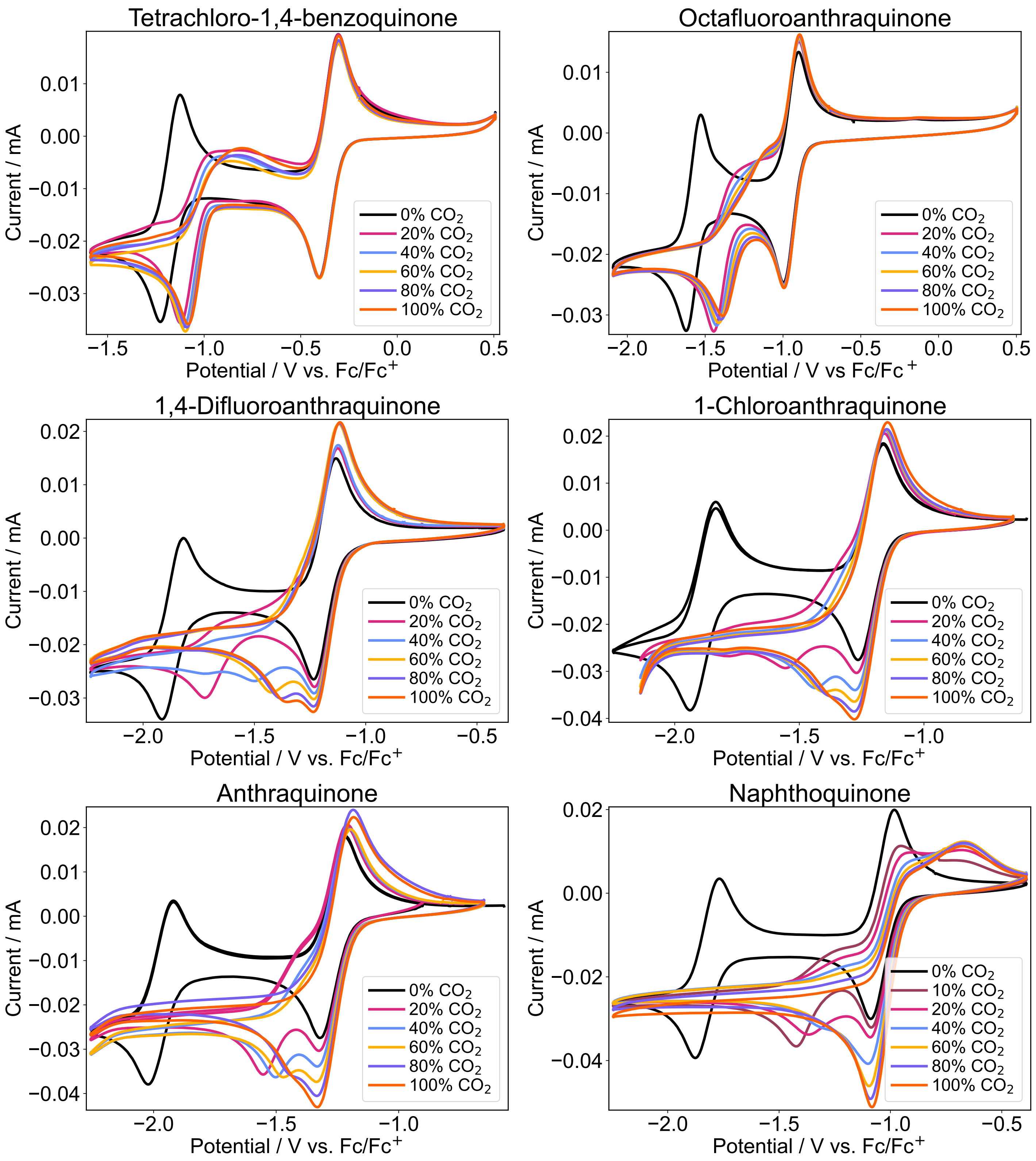

Figure S1: The CV results for various quinones at various CO_2 concentrations. In each case the third (last) cycle of the experiment at 20 mVs^{-1} is shown, and used for all further analysis.

S3.3 CV Results at 100 mVs^{-1}

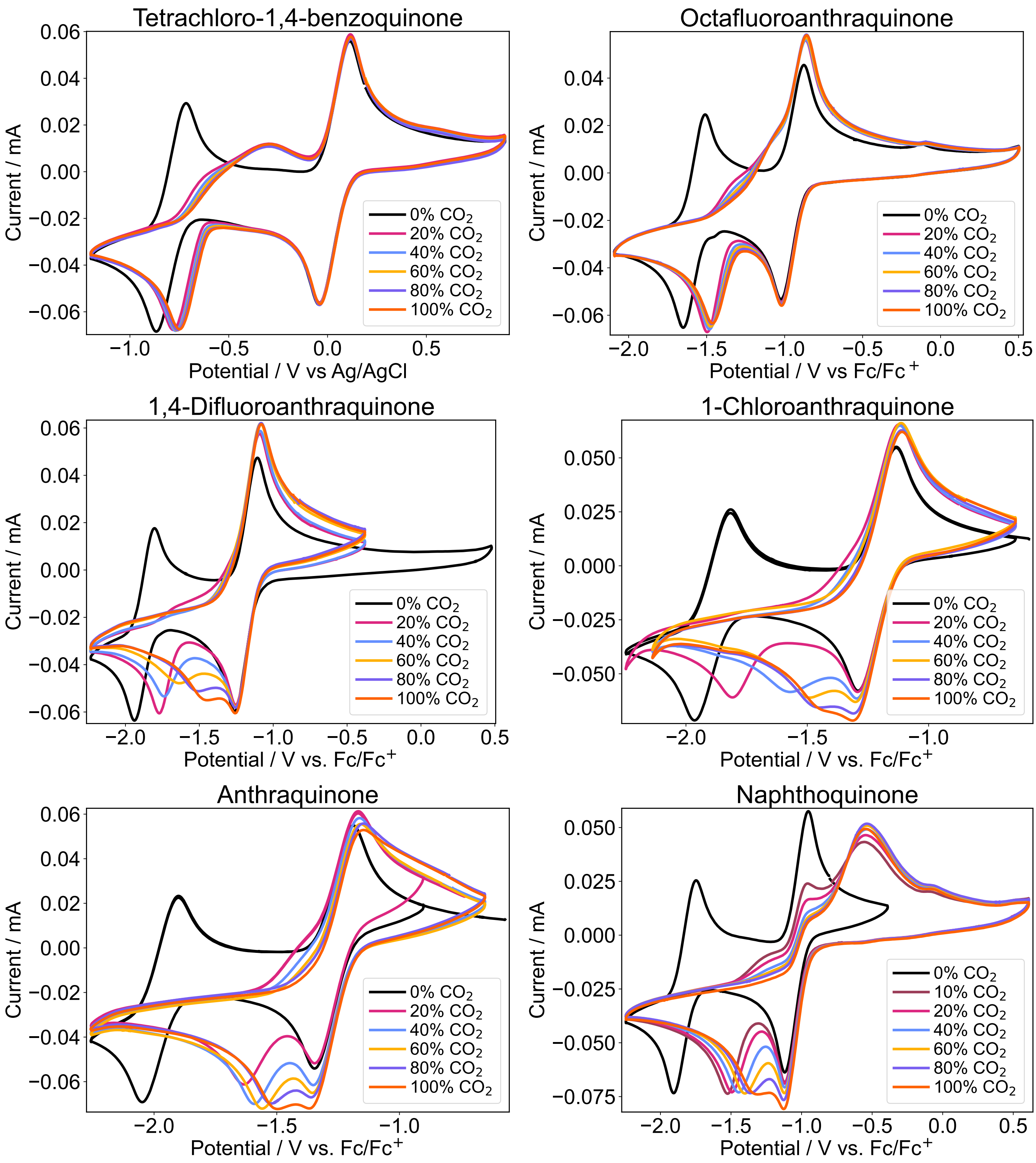

Figure S2: The CV results for various quinones at various CO_2 concentrations. In each case the third (last) cycle of the experiment at 100 mVs^{-1} is shown, and used for all further analysis.

S3.4 Derivation of log$K$ from Shift in Second Reduction Wave at Variable CO_2 Concentrations

The derivation relies on the following equilibrium, Eq. (S6), where Q^{2-} corresponds to a quinone dianion. n can be thought of as the average number of CO_2 molecules each quinone dianion captures on average. It is not a requirement that n be an integer.

$$Q^{2-} + nCO_2 \xrightleftharpoons{K} Q(CO_2)_n^{2-} \tag{S6}$$

K is the general CO_2 binding constant for the specified quinone, Eq. (S7). c° is the standard concentration which we define as 1 mol dm^{-3}.

$$K = \frac{[Q(CO_2)_n^{2-}]}{[Q^{2-}][CO_2]^n} \times (c^\circ)^n \tag{S7}$$

For the next step in the derivation, the Nernst equation is used to relate the measured potential under N_2, E, to the relative concentrations of the quinone dianion (Q^{2-}) and the semiquinone ($Q^{\bullet-}$), Eq. (S8).

$$E = E^{\circ}_{½,N_2} - \frac{RT}{F} \ln\left(\frac{[Q^{2-}]}{[Q^{\bullet-}]}\right) \tag{S8}$$

Next, a new quantity, $[Q^{2-}]_{\text{Total}}$, is defined which corresponds to the total concentration of dianion species in solution, Eq. (S9). This can be written in terms of the quinone dianion concentration, general CO_2 binding constant, value of n and the CO_2 concentration in solution, Eq. (S10).

$$[Q^{2-}]_{\text{Total}} = [Q^{2-}] + [Q(CO_2)_n^{2-}] \tag{S9}$$

$$[Q^{2-}]_{\text{Total}} = [Q^{2-}](1 + K[CO_2]^n) \tag{S10}$$

The next step is assuming that the half-wave potential in the presence of CO_2, $E^{\circ}_{½,CO_2}$, occurs at the potential when $[Q^{\bullet-}] = [Q^{2-}]_{\text{Total}}$. This condition can be combined with Eq. (S8) to obtain the following expressions for $E^{\circ}_{½,CO_2}$.

$$E^{\circ}_{½,CO_2} = E^{\circ}_{½,N_2} - \frac{RT}{F} \ln\left(\frac{[Q^{2-}]}{[Q^{2-}](1 + K[CO_2]^n}\right) \tag{S11}$$

$$E^{\circ}_{½,CO_2} = E^{\circ}_{½,N_2} + \frac{RT}{F} \ln(1 + K[CO_2]^n) \tag{S12}$$

The shift in redox potential, $\Delta E^{\circ}_{½}$, is defined, Eq. (S13), and using Eq. (S11) it can be expressed in terms of the thermodynamic parameters, n, the CO_2 concentration, and K, Eq. (S14).

$$\Delta E^{\circ}_{½} = E^{\circ}_{½,CO_2} - E^{\circ}_{½,N_2} \tag{S13}$$

$$\Delta E^{\circ}_{½} = \frac{RT}{F} \ln(1 + K[CO_2]^n) \tag{S14}$$

The next approximation which is generally made is that $K[CO_2]^n$ is much greater than 1. In this case, the equation for $\Delta E^{\ominus}_{1/2}$ can be simplified, Eq. (S15).

$$\Delta E^{\ominus}_{1/2} \approx \frac{RT}{F} \left(\ln K + n \ln[CO_2] \right) \tag{S15}$$

When Eq. (S15) is rearranged, Eq. (S16), it becomes clear that a plot of $(F/RT)\Delta E^{\ominus}_{1/2}$ against $[CO_2]$ should yield a straight line with gradient equal to n and a y-intercept equal to $\ln K$.

$$\frac{F}{RT}\Delta E^{\ominus}_{1/2} = n \ln[CO_2] + \ln K \tag{S16}$$

In practice, and in this work, $\Delta E^{\ominus}_{1/2}$ is measured from cyclic voltammetry as the shift in the second reduction peak. The oxidation peak is not generally used as it is significantly broader and less well-defined than the reduction peak once CO_2 is introduced.

The concentration of CO_2 is calculated using Henry's law,

$$[CO_2] = P_{CO_2} H_{CO_2} \tag{S17}$$

where $[CO_2]$ is the CO_2 concentration, P_{CO_2} is the partial pressure of CO_2 in the headspace, and H_{CO_2} is the Henry's constant for CO_2 in the specified solvent at a specified temperature. In DMSO at 25°C, it has been reported[15] that $H_{CO_2} = 728$ Pa m^3 mol^{-1}. It is then assumed that $P_{CO_2} = 1$ atm $\times \frac{\%CO_2}{100}$ such that $[CO_2]$ is a maximum when purging with 100% CO_2 and that the maximum solubility is 0.139 M.

In this work it is assumed that the Henry constant for CO_2 in DMSO does not change after the addition of electrolyte and quinone.

S3.5 Shift vs CO_2 Concentration Plots at 20 mVs^{-1}

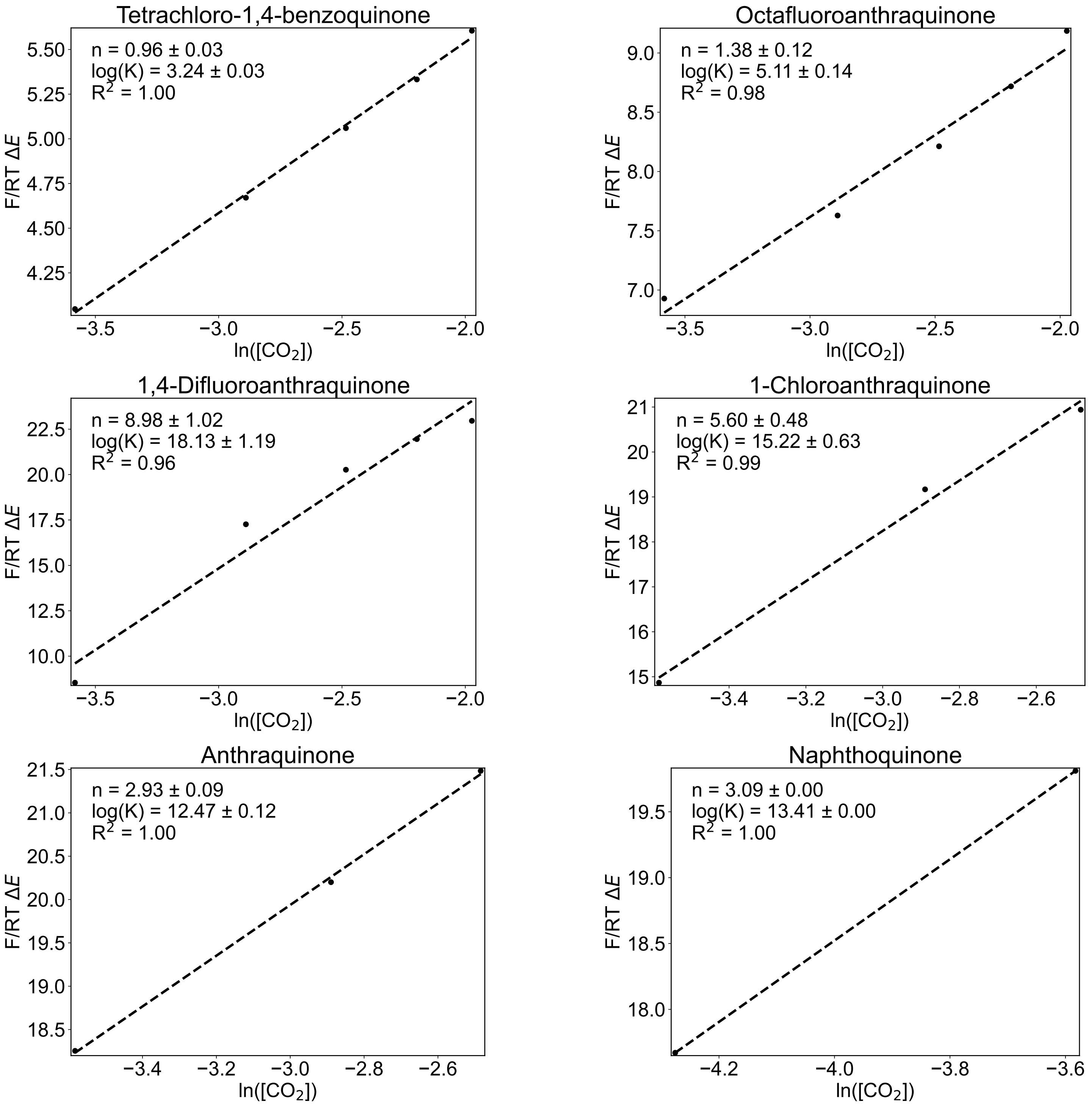

Figure S3: Plots to determine n and $\log(K)$ from the CV results in Fig. S1 at 20 mVs^{-1}. For each plot linear regression is performed to obtain a line of best fit, which is then also plotted. From the line of best fit, n and $\log(K)$ are calculated and errors calculated as the standard errors in the linear regression analysis. The R^2 for the fit is also provided. For 1-chloroanthraquinone, anthraquinone, and naphthoquinone, at higher CO_2 concentrations the second reduction peak is no longer well resolved, hence these plots contain fewer data points.

S3.6 Shift vs CO_2 Concentration Plots at 100 mVs^{-1}

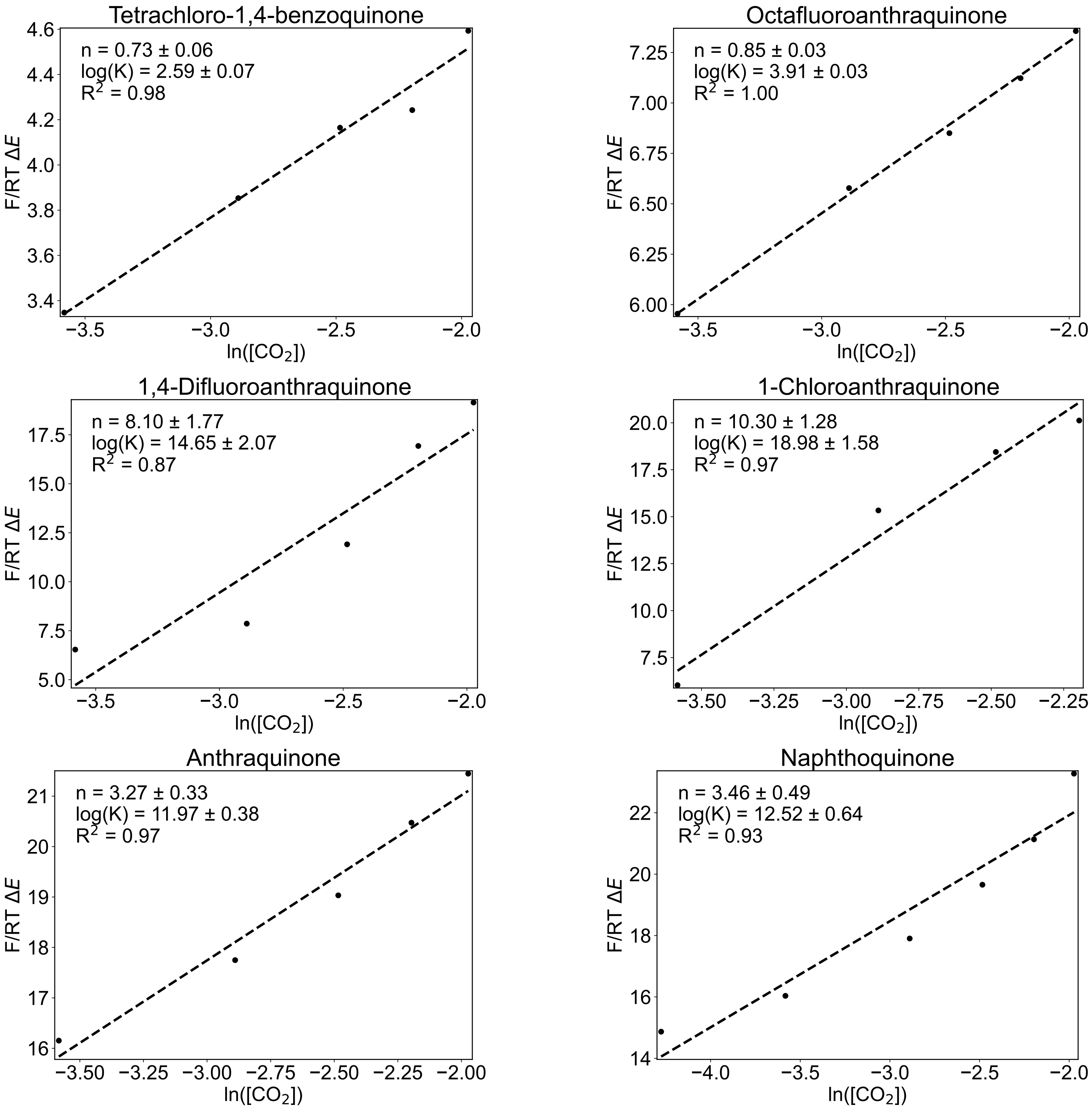

Figure S4: Plots to determine n and $\log(K)$ from the CV results in Fig. S2 at 100 mVs^{-1}. For each plot linear regression is performed to obtain a line of best fit, which is then also plotted. From the line of best fit, n and $\log(K)$ are calculated and errors calculated as the standard errors in the linear regression analysis. The R^2 for the fit is also provided.

S3.7 Summary of Experimental Values for log$K$ and E_{2e}

S3.7.1 20 mV s^{-1}

Quinone	E_{2e} / V	log$K$	n
Tetrachloro-1,4-benzoquinone	-0.766	3.24 ± 0.03	0.96 ± 0.03
Octafluoroanthraquinone	-1.262	5.11 ± 0.14	1.38 ± 0.12
1,4-Difluoroanthraquinone	-1.526	18.1 ± 1.2	9.0 ± 1.0
1-Chloroanthraquinone	-1.550	15.2 ± 0.7	5.6 ± 0.5
Anthraquinone	-1.620	12.47 ± 0.13	2.93 ± 0.09
Naphthoquinone	-1.429	13.41 ± N.A.	3.1 ± N.A.

Table S1: Summary of experimental values for the two electron-redox potential and the log of the general binding constant to CO_2 for a variety of quinones based off analysis of cyclic voltammograms at 20 mVs^{-1}. For naphthoquinone linear regression was performed on only two data points so no errors are reported.

S3.7.2 100 mV s^{-1}

Quinone	E_{2e} / V	log$K$	n
Tetrachloro-1,4-benzoquinone	-0.766	2.59 ± 0.07	0.73 ± 0.06
Octafluoroanthraquinone	-1.262	3.91 ± 0.03	0.85 ± 0.03
1,4-Difluoroanthraquinone	-1.526	15 ± 2	8.1 ± 1.8
1-Chloroanthraquinone	-1.550	19.0 ± 1.6	10.3 ± 1.3
Anthraquinone	-1.620	12.0 ± 0.4	3.3 ± 0.3
Naphthoquinone	-1.429	12.5 ± 0.7	3.5 ± 0.5

Table S2: Summary of experimental values for the two electron-redox potential and the log of the general binding constant to CO_2 for a variety of quinones based off analysis of cyclic voltammograms at 100 mVs^{-1}.

S3.8 Isolating Second Reduction Peak

For 1,4-difluoroanthraquinone, 1-chloroanthraquinone, anthraquinone, and naphthoquinone, as the CO_2 concentration was increased, the second reduction peak shifted towards the first and this at times made it impossible to define where the actual peak was located, Figs. S1 and S2. The result in some cases was that only a few points could be used for calculating logK, Figs. S3 and S4, and that the actual peak locations were likely distorted, introducing additional error. We therefore aimed to isolate the second reduction peak to obtain more accurate values of n and $\log(K)$.

S3.8.1 Method

Note first that for the quinones tetrachloro-1,4-benzoquinone and octafluoroanthraquinone the more positive (first) reduction wave has constant shape and intensity, Fig. S1. We therefore assume that the same is true for the other quinones, and that apparent increases in intensity or change in shape are due to overlap with the more negative (second) reduction wave. To isolate the second reduction wave, we used the CV under N_2 at 20 mVs^{-1} as a reference, and subtracted it from the CVs with CO_2 present, also at 20 mVs^{-1}, effectively removing the first reduction wave.

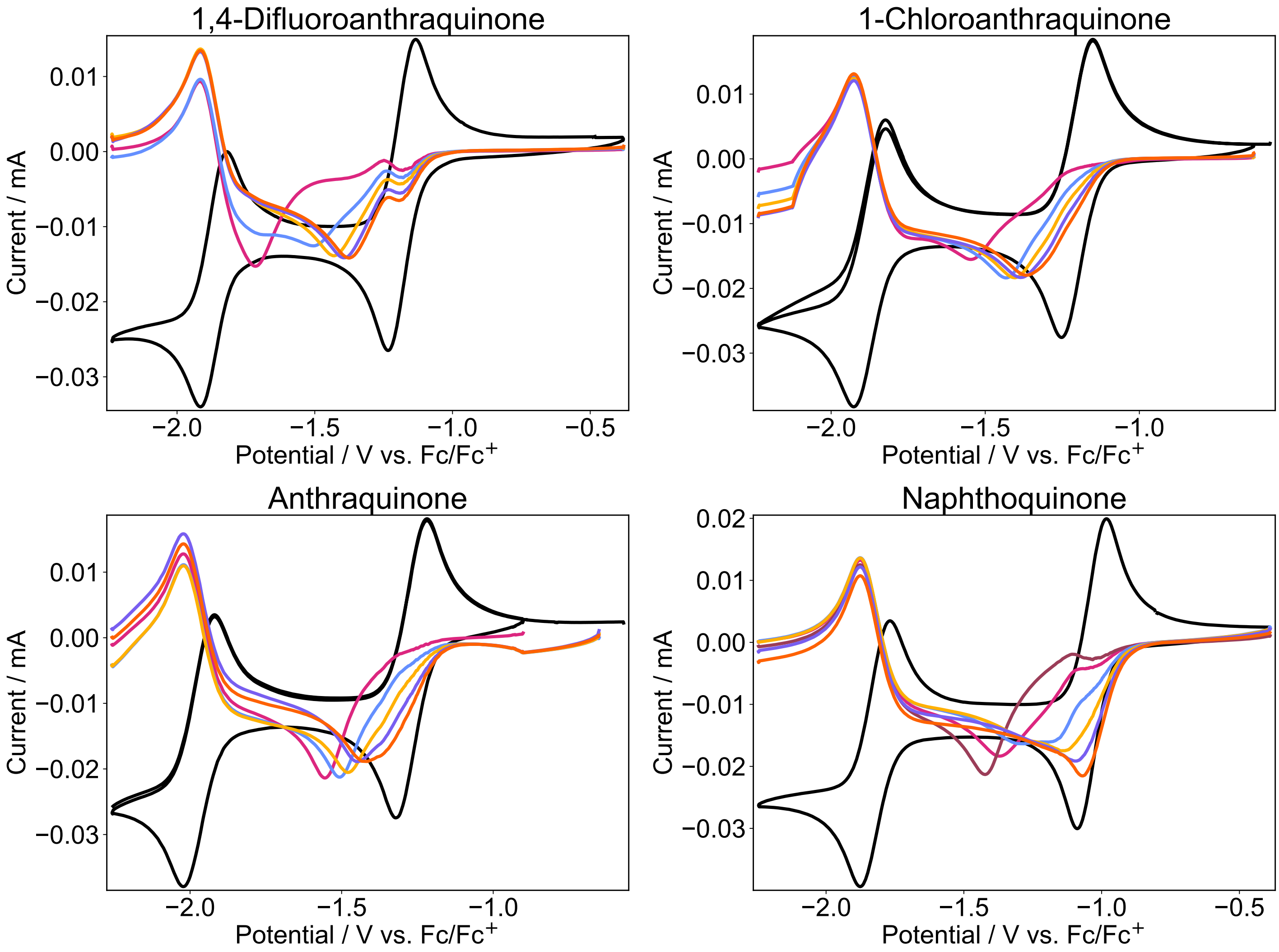

Figure S5: CV responses (20 mVs^{-1}) for four strongly binding quinones at various CO_2 concentrations, after subtraction of the CV response under N_2. In each plot the reference CV is also included for comparison.

We then plotted the current signal, with the reference subtracted, and treated the observed remaining wave as the form of the true second reduction wave, Fig. S5.

S3.8.2 Discussion on CV Results

The method appeared to work well, with second reduction peaks now resolved at all CO_2 concentrations for 1-chloroanthraquinone and also for anthraquinone. For 1,4-difluoroanthraquinone although the peaks appear resolved note that at 40% CO_2 concentrations there appear to be two reduction peaks, similar to what was also observed in Fig. S1. This may indicate that for this quinone the electrochemistry is more complicated and not well described by our analysis model.

In the case of naphthoquinone, it is assumed that after 40% CO_2, the second reduction wave completely merges with the first, and then even though there appears to be well defined peaks, these are simply due to increase in intensity from a two-electron reduction, thus they cannot be used as positions of the one-electron reduction.

S3.8.3 Plots and Discussion of New Values for n and $\log(K)$

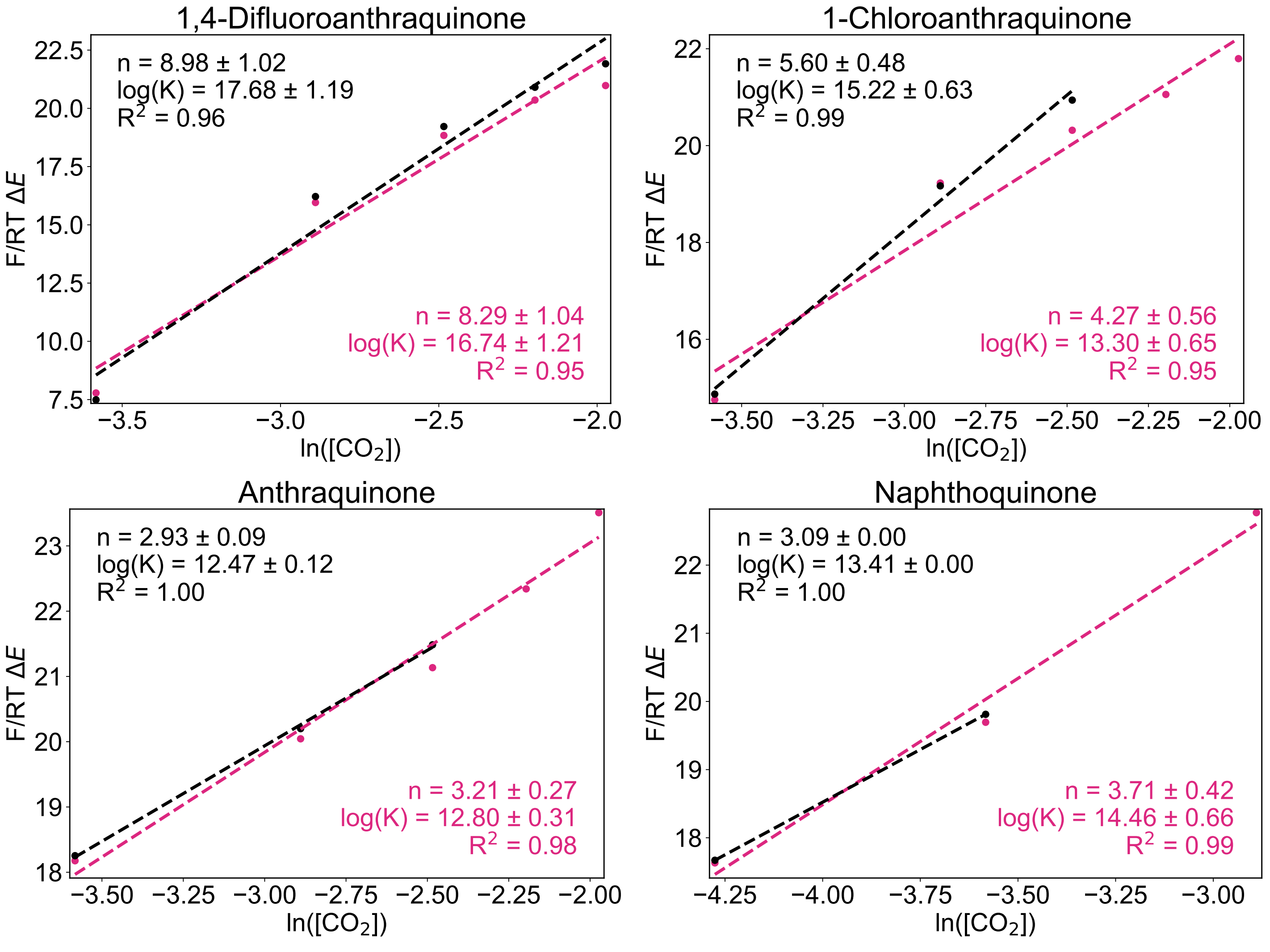

Figure S6: Plots of shift in second reduction peak vs log of CO_2 concentration for four stringly binding quinones. In black are the data points from direct analysis of the CVs at 20 mVs^{-1}. In pink are the data points obtained from first removing the N_2 reference CV. Linear regression is performed on all data sets to obtain a line of best fit, from which n, $\log(K)$ and R^2 are obtained.

Using the new, corrected values of ΔE, obtained from the Fig. S5, updated values for n and $\log(K)$ could be obtained, Fig. S6.

For anthraquinone and 1-chloroanthraquinone, we were able to add an additional two data points to the plots. For anthraquinone, the extra points fit extremely well and we believe this is good evidence that the subtraction of a reference CV method appears to be reasonable.

For naphthoquinone, only one extra data point was able to be added, however with three points, errors can then be obtained from linear regression analysis.

For 1,4-difluoroanthraquinone, no extra data points were added, however at higher CO_2 concentrations the shift in reduction potential was found to be smaller than previously estimated, and this led to a small reduction in the calculated value of logK.

S3.8.4 Tabulated Corrected Experimental Values for log$K$ and E_{2e}

Quinone	E_{2e} / V	log$K$	n	corrected log$K$	corrected n
Tetrachloro-1,4-benzoquinone	-0.766	3.24 ± 0.03	0.96 ± 0.03		
Octafluoroanthraquinone	-1.262	5.11 ± 0.14	1.38 ± 0.12		
1,4-Difluoroanthraquinone	-1.526	18.1 ± 1.2	9.0 ± 1.0	16.7 ± 1.2	8.3 ± 1.0
1-Chloroanthraquinone	-1.550	15.2 ± 0.7	5.6 ± 0.5	13.3 ± 0.7	4.3 ± 0.6
Anthraquinone	-1.620	12.47 ± 0.13	2.93 ± 0.09	12.8 ± 0.3	3.2 ± 0.3
Naphthoquinone	-1.429	13.41 ± N.A.	3.1 ± N.A.	14.5 ± 0.7	3.7 ± 0.4

Table S3: Summary of experimental values for the two electron-redox potential and the log of the general binding constant to CO_2 obtained using purely the observed shift in reduction potential and the corrected values which are obtained through isolation of the second reduction peak via subtraction of the reference CV under N_2.

S3.9 Validation of Reference Subtraction Method

To validate this method of subtracting the CV obtained under N_2 from the subsequent CVs which are exposed to CO_2, a short study with simulated CVs was conducted.

S3.9.1 CV Simulation Methodology

The following CV simulations were generated using ECSimulate which is in-house simulation software available at https://github.com/JackScottTaylor/ECSimulate.

The simulation modelled diffusion using a Crank-Nicholson formulation. The electrode was modelled by enforcing the flux of species to the surface to match the fluxes calculated via Butler-Volmer kinetics, at the other boundary, the concentrations of all species were set equal to their initial bulk concentrations. An exponentially expanding spatial grid was used such that 100 points were considered, with a minimum spacing of 1×10^{-4} cm and a total simulation width of 0.1 cm. The time step was set to 10^{-3} s and the scan rate to 20 mVs^{-1}. In each case one scan was modelled, starting at +0.5 V and going to a minimum potential of -1.0 V. All CVs were modelled at 25°C.

All species were modelled with a diffusion coefficient of $3.5 \times 10^{-6} cm^2 s^{-1}$. The starting concentration of the most oxidised species was set to 5 mM and all others to zero. Each redox reaction was modelled using Butler-Volmer kinetics, with a rate constant of 1 cm s^{-1} (corresponding to fast and reversible electron transfer) and a transfer coefficient equal to 0.5.

Reference Scan: The reference was modelled as two one-electron redox processes, the first at 0.0 V and the second at -0.75 V.

Overlapping Scan: The second CV, used to represent a quinone CV after the addition of CO_2, was modelled as again two one-electron redox processes. As before the first redox potential was set to 0.0 V, however this time the second redox potential was set to -0.1 V, such that it would overlap significantly with the more positive redox wave.

"True" Scan: The final CV to be simulated was a single redox wave at -0.1 V. This was simulated to compare the reference-subtracted CV to.

S3.9.2 Results

For a single-electron reversible redox wave at 25°C, the peak of the reduction wave should be 28.5 mV more negative than the standard redox potential.[16] Therefore for the redox even modelled at -0.1 V, the reduction peak should occur at -0.1285 V, which is the value obtained by the single redox-wave simulation, Fig. S7 yellow.

It should be first noted that the reduction peak value obtained directly from the overlapping signal is 9.7 mV positive of the ideal value, Fig. S7 red. Relating this model to the CVs observed for quinones under the presence of CO_2, this would translate in to the reduction peak shift being overestimated, which would lead to overestimates of n and $\log K$.

When the reference CV is subtracted from the simulated overlapping CV signal, Fig. S7 blue, the resulting signal almost exactly matches the single redox-wave simulation around the potentials of interest. This significantly improves the obtained value of the observed reduction peak, which is now only 0.6 mV positive of the expected value.

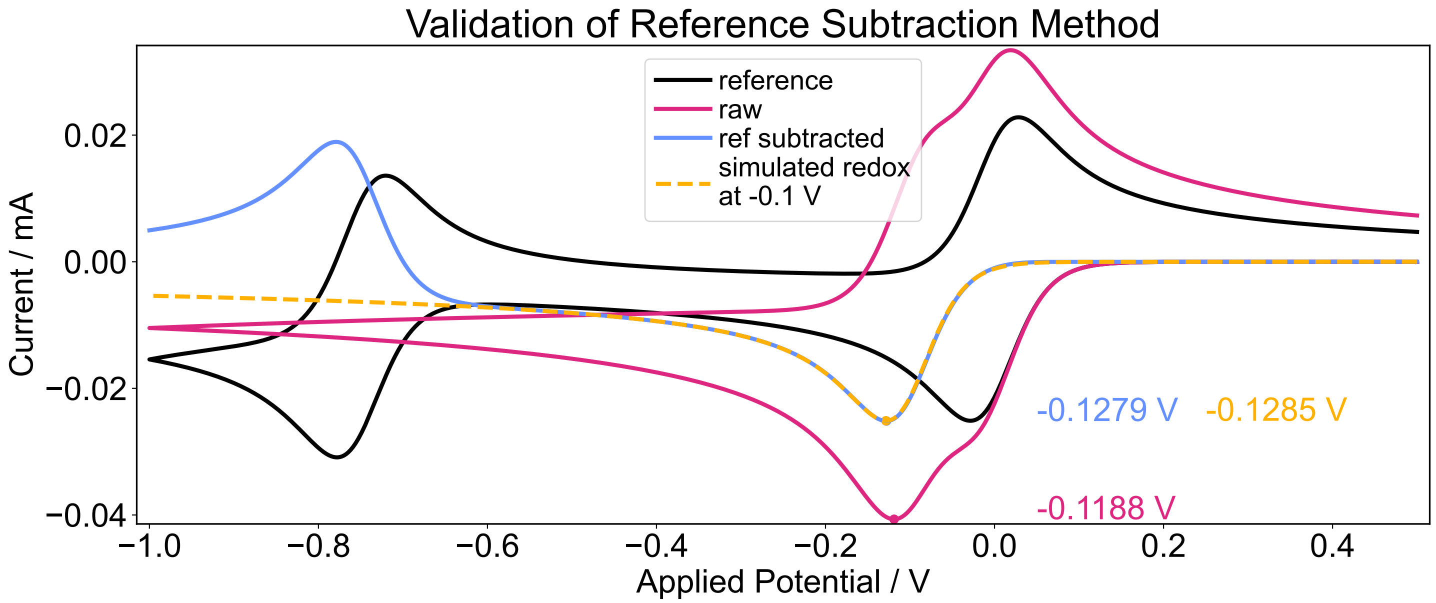

Figure S7: Various CV simulations to explore the validity of the reference subtraction method. In black is the simulated CV corresponding to two well separated redox waves and is used as the reference. In pink is a simulated CV with significantly overlapping redox waves, with the more positive redox wave identical to the black CV. In blue is the result of subtracting the forward sweep of the reference CV from the forward sweep of the overlapping CV. In yellow is the forward sweep of a simulated CV containing only a single redox event at -0.1 V. The potentials at which the minimum current occurs for the "raw" overlapping CV, the reference-subtracted CV, and the simulates single-redox CV are also given in their corresponding colours.

In conclusion, when there is overlap of redox waves, and it can be assumed that the more positive redox wave has not been altered, subtraction of the reference CV allows for a more accurate value of the reduction peak potential to be obtained. In this short CV simulation study we have observed that using the non-subtracted peak value will likely lead to overestimations of n and $\log K$, therefore subtraction of the reference CV is both justified, and should lead to more accurate results.

S3.10 Further Consideration of Double Peak Behaviour

It was noted that in the CVs of 1,4-difluoroanthraquinone and 1-chloroanthraquinone, for some CO_2 concentrations there appeared to be an additional reduction peak. This does not fit with the model used to analyse the CVs and may have led to erroneous values of n and $\log K$.

To further investigate this matter, the CV experiments for 1,4-difluoroanthraquinone were repeated at scan rates of 200, 100, 50, 20 and 10 mV s^{-1}. In this case the potentials are referenced against the Ag/AgCl reference electrode used.

The use of different scan rates, Figs. S9 and S10, revealed some fascinating behaviour. There are broadly two regions where there are defined shifted reduction peaks when CO_2 is introduced, at approximately -1.4 V and -1.1 V. Peaks roughly between these two regions appear broadened.

We note that the peaks appear closer to the -1.4 V region when there is a combination of lower CO_2 concentration and faster scan rate. For high CO_2 concentration and slow scan rate the peaks always appear relatively sharp and in the -1.1 V region. From these results we hypothesise that there is a slow process responsible for the shift from the -1.4 V region to the -1.1 V region, and a faster process responsible for the first minor shift to the -1.4 V region.

The two processes can be taken to be the two independent CO_2 capture steps, Fig. S8, where we predict that the second CO_2 capture is much slower than the first.

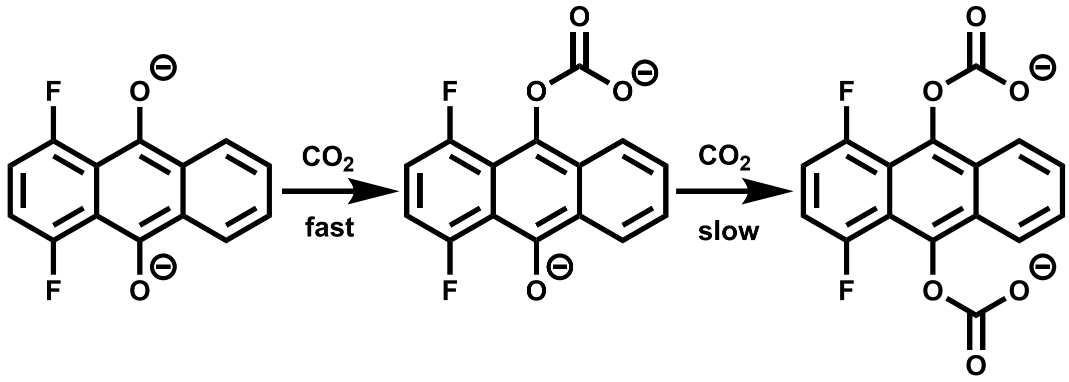

Figure S8: The hypothesised mechanism for CO_2 capture by the 1,4-difluoroanthraquinone dianion to explain the observed behaviour in cyclic voltammetry.

The second CO_2 capture being slow would explain the CO_2 dependence observed on which region the shifted peak presents in. The scan rate dependence is explained as at high scan rate there is not time for the second capture to happen, so over the timescale of the fast CV experiment, it appears insignificant.

These results imply that when the shifted peak appears in the -1.4 V region, that the CV is being recorded too fast and the effect of the second capture is not being observed.

To be consistent with the other CVs used for calibration purposes and to be in line with the standard practises for the field, we use the CVs recorded at 20 mV s^{-1} to calculate the binding constant. As there is significant overlap with the first redox wave at 20 mV s^{-1} we apply the reference-subtraction method used previously and subtract the first reduction wave from the CVs to obtain a more accurate value for the position of the second peak.

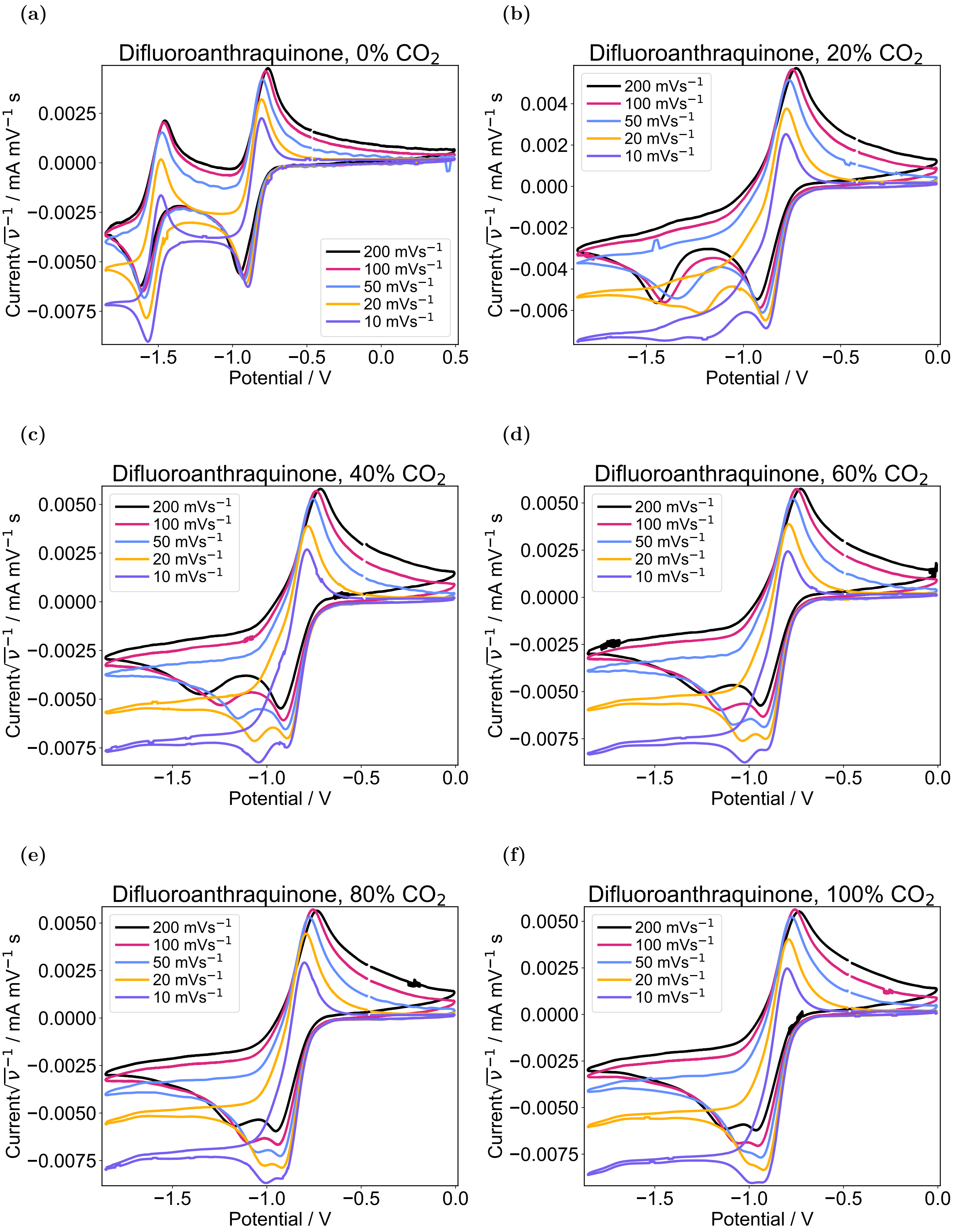

Figure S9: Cyclic voltammograms of 1,4-difluoroanthraquinone (5 mM) in DMSO with 0.1 M $TBAPF_6$ supporting electrolyte at 25°C. Each subfigure corresponds to a different percentage of CO_2 in the purging gas. The current response is scaled by the square root of the applied scan rate.

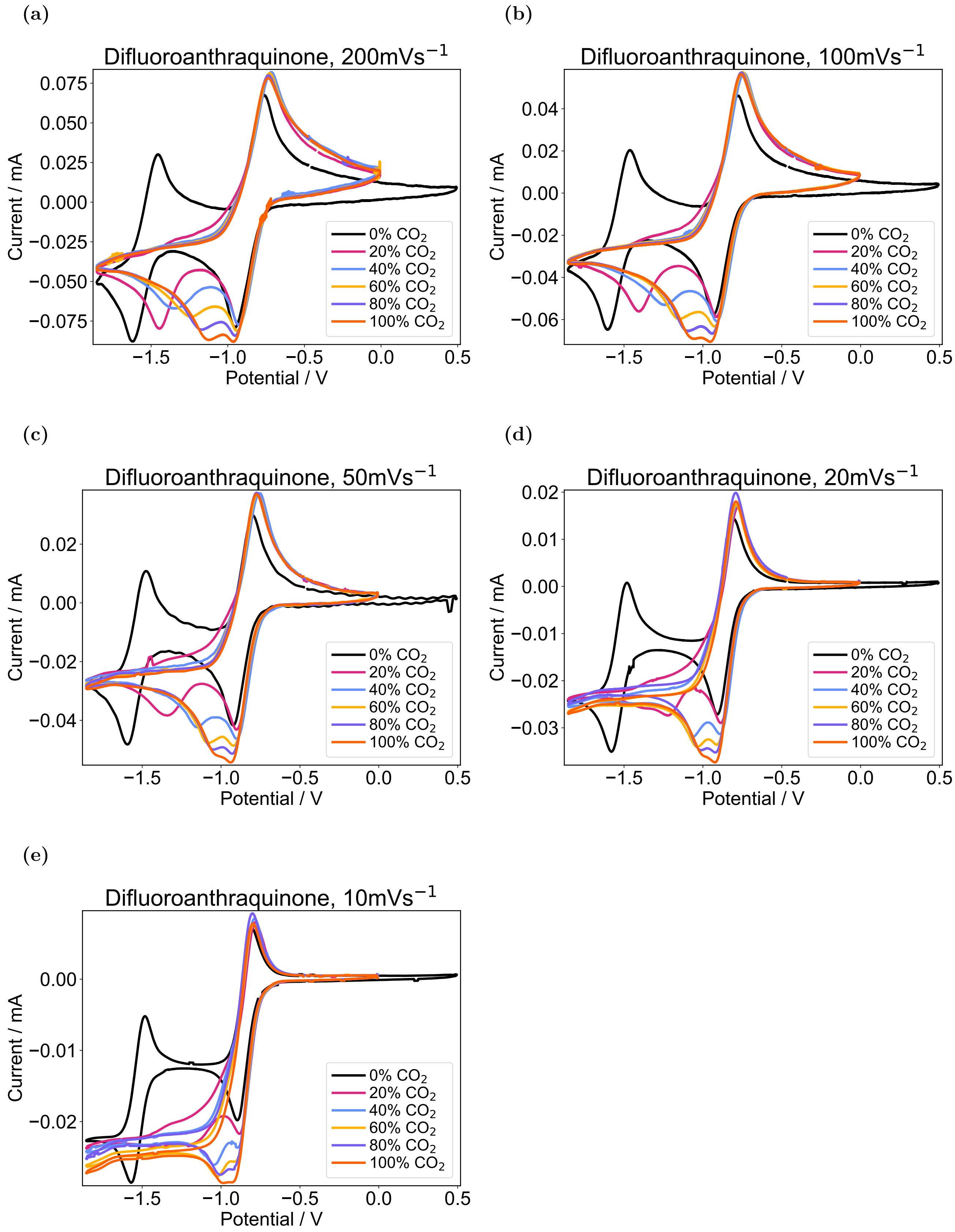

Figure S10: Cyclic voltammograms of 1,4-difluoroanthraquinone (5 mM) in DMSO with 0.1 M $TBAPF_6$ supporting electrolyte at 25°C. Each subfigure corresponds to a different scan rate used in obtaining the CVs. The different colours correspond to different percentages of CO_2 used in the purging gas.

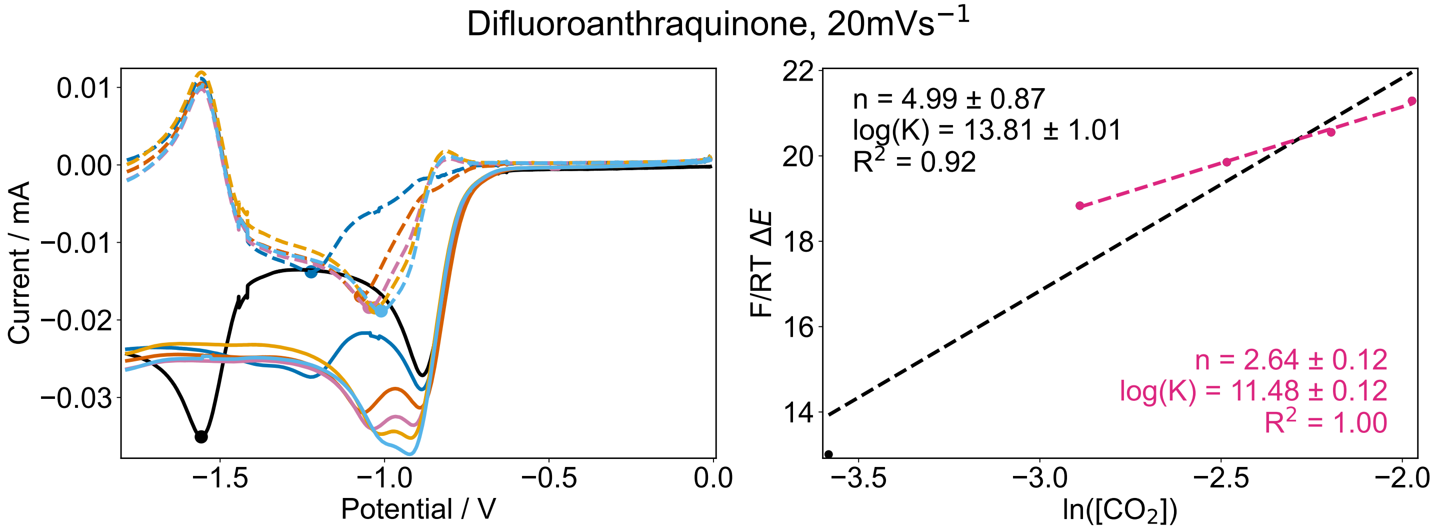

Figure S11: Left: In bold are the current responses to the forward sweep of the cyclic voltammogram of 1,4-difluoroanthraquinone (5 mM) in DMSO with 0.1 M $TBAPF_6$ supporting electrolyte at 25°C and 20 mV s^{-1} purged with various concentrations of CO_2. The dashed lines correspond to the forward-sweeps with non-zero CO_2, with the response under pure N_2 (black) subtracted. The position of the second reduction peak is then taken as the position of the most negative current in the subtracted response (labelled with a dot). **Right:** Analysis of the measured reduction peak shifts under various CO_2 concentrations both including (black) and excluding (pink) the measured shift for 20% CO_2.

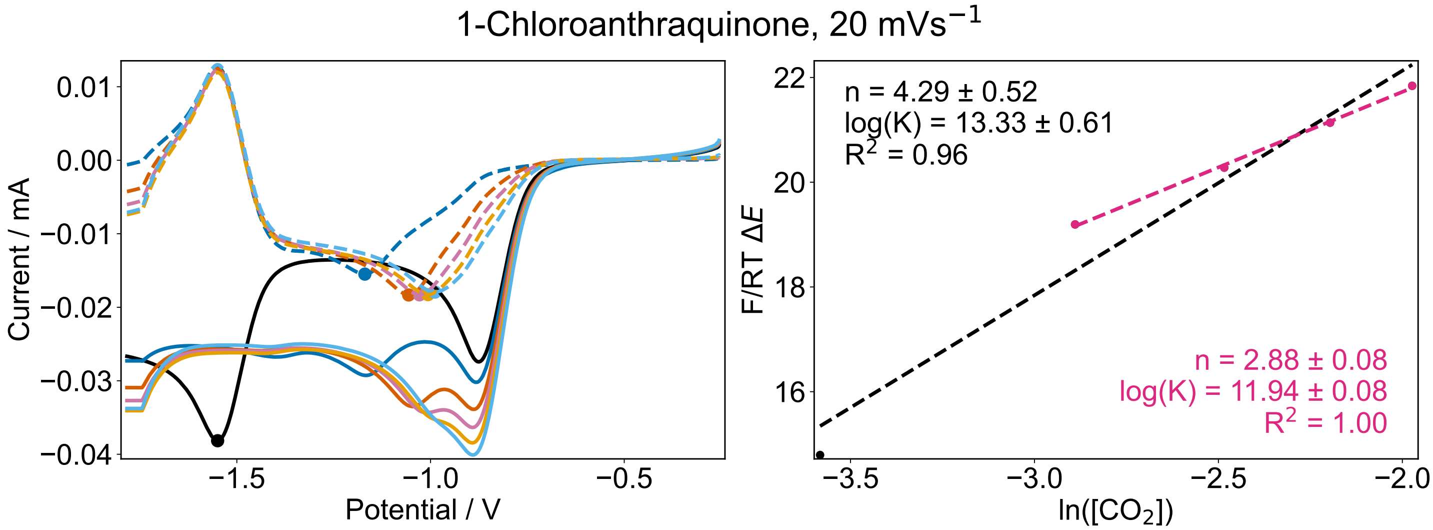

Figure S12: Left: In bold are the current responses to the forward sweep of the cyclic voltammogram of 1-chloroanthraquinone (5 mM) in DMSO with 0.1 M $TBAPF_6$ supporting electrolyte at 25°C and 20 mV s^{-1} purged with various concentrations of CO_2. The dashed lines correspond to the forward-sweeps with non-zero CO_2, with the response under pure N_2 (black) subtracted. The position of the second reduction peak is then taken as the position of the most negative current in the subtracted response (labelled with a dot). **Right:** Analysis of the measured reduction peak shifts under various CO_2 concentrations both including (black) and excluding (pink) the measured shift for 20% CO_2.

We noted that the peak at 20% CO_2 appeared in the middling region, hence by our hypothesis the effect of the second CO_2 capture is not accurately observed at this CO_2 concentration. We considered the values extracted if the 20% point is considered and if not. We found that by using only data points where we believe the second CO_2 capture is being accurately measured, that values of n and $\log K$ more in line with the results for the previous quinones are obtained.

As some double-peak character was also observed in the 1-chloroanthraquinone CVs, we believe that the situation is similar, where the second CO_2 capture is slow and therefore not always significant depending on the CO_2 concentration and scan rate. We therefore reanalysed the data and determined that the shift under 20% CO_2 should be excluded when extracting n and $\log K$.

S3.10.1 Tabulated Fully Corrected Experimental Values for log$K$ and E_{2e}

Quinone	E_{2e} / V	log$K$	n
Tetrachloro-1,4-benzoquinone[1]	-0.766	3.24 ± 0.03	0.96 ± 0.03
Octafluoroanthraquinone[1]	-1.262	5.11 ± 0.14	1.38 ± 0.12
1,4-Difluoroanthraquinone[3]	-1.526	11.48 ± 0.12	2.64 ± 0.12
1-Chloroanthraquinone[3]	-1.550	11.94 ± 0.08	2.88 ± 0.08
Anthraquinone[2]	-1.620	12.8 ± 0.3	3.2 ± 0.3
Naphthoquinone[2]	-1.429	14.5 ± 0.7	3.7 ± 0.4

[1] n and log$K$ derived from peak shift observed in CV without further manipulation.
[2] n and log$K$ derived from peak shift observed after subtracting reference CV from measured response.
[3] n and log$K$ derived from peak shift observed after subtracting reference CV from measured response and excluding shifts which are not considered to be capturing effect of second CO_2 capture.

Table S4: Summary of experimental values for the two electron-redox potential and the log of the general binding constant to CO_2. Each quinone is also labelled with the method used to obtain the reported values of log$K$ and n.

S4 Calibration of Computational Results

Using the experimental values of log(K) and E_{2e} found from CV analysis, Table S3, the computational results could be calibrated. For each of the six quinones investigated experimentally, the log(K) and E_{2e} values were computationally predicted using the methods described in Section S2 and then compared to the experimental results, Fig. S13

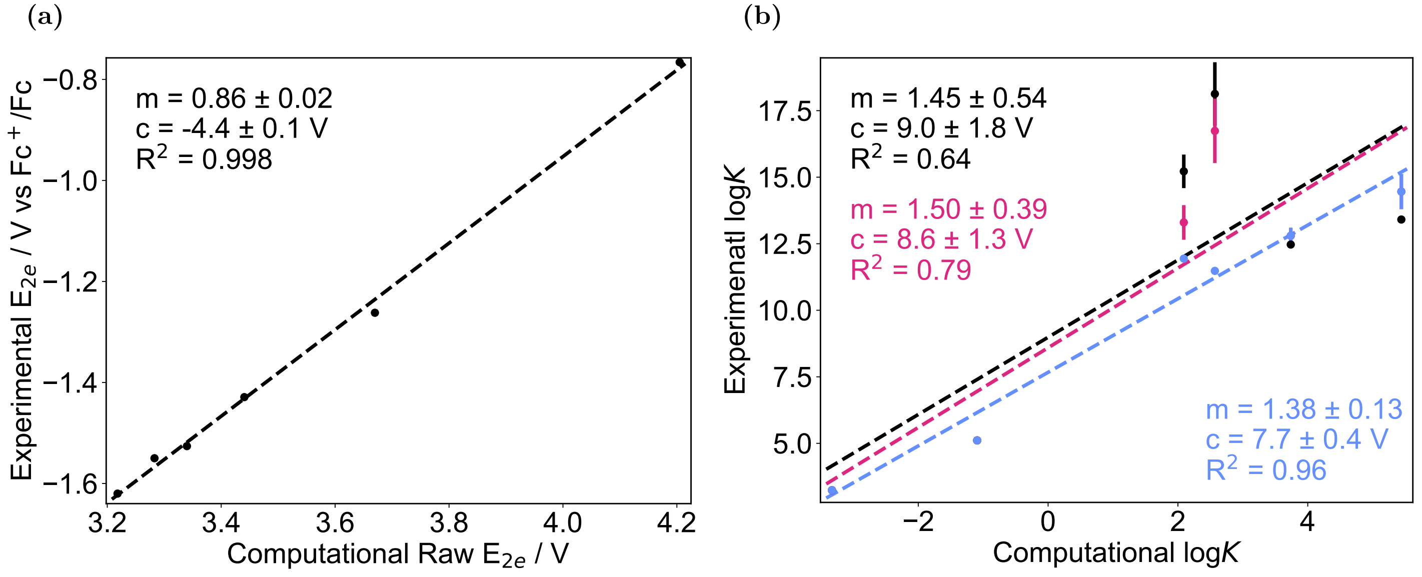

Figure S13: (a): Comparison of experimental to computational results for E_{2e}. Linear regression analysis is used to obtain a line of best fit, as well as a value for R^2. All parameters also included in figure. **(b):** Comparison of experimental to computational results for log(K). Black points correspond to the experimental values from direct CV analysis, pink corresponds to the results obtained after applying the reference-subtraction procedure on the more strongly binding CVs, and the blue points correspond to the final results after reanalysis of the CVs containing some double reduction peak character. All data sets have had linear regression applied to obtain lines-of-best-fit and the calculated parameters are included in the figure.

S4.1 Discussion of Calibration Results

The correlation between experimental and computational results for E_{2e}, Fig. S13a, is exceptional and indicates that the computational methods can be used to predict accurate two-electron redox potentials.

For the calibration of log(K) values, Fig. S13b, the correlation between computational and experimental initially appeared much poorer. After refinement of the ΔE values using the reference-subtraction method described earlier, the line of best fit interestingly did not significantly change, however the value of R^2 increased significantly.

It is worth briefly discussing the very large (> 3) values of n obtained for some of the quinones. Values of n greater than two are not physical with the mechanism considered for CO_2 capture, however as of writing, to the best of the authors' knowledge, there are no superior methods for calculating these binding constants.

Generally the authors believe that these unphysical values are due to slow CO_2 release by the dianion such that the concentrations at the electrode surface are not at equilibrium. By performing all measurements at 20 mVs^{-1} this therefore introduces a systematic error in extracting these thermodynamic values. It is the authors intention to prepare a future publication further exploring the consequences and origins of these unphysical values.

S5 Tetrachloro-1,2-benzoquinone

Cyclic voltammograms of tetrachloro-1,2-benzoquinone were taken in the same manner as the previous six quinones, except that in this case the quinone was added once the correct temperature of 25°C was obtained. In the standard procedure, the temperature would often exceed 35°C before cooling to the correct temperature. Of the seven quinones, tetrachloro-1,2-benzoquinone is the only one where it is recommended to store in a refrigerator. Therefore, we wanted to minimise exposure of the quinone to high temperatures, to prevent possible decomposition.

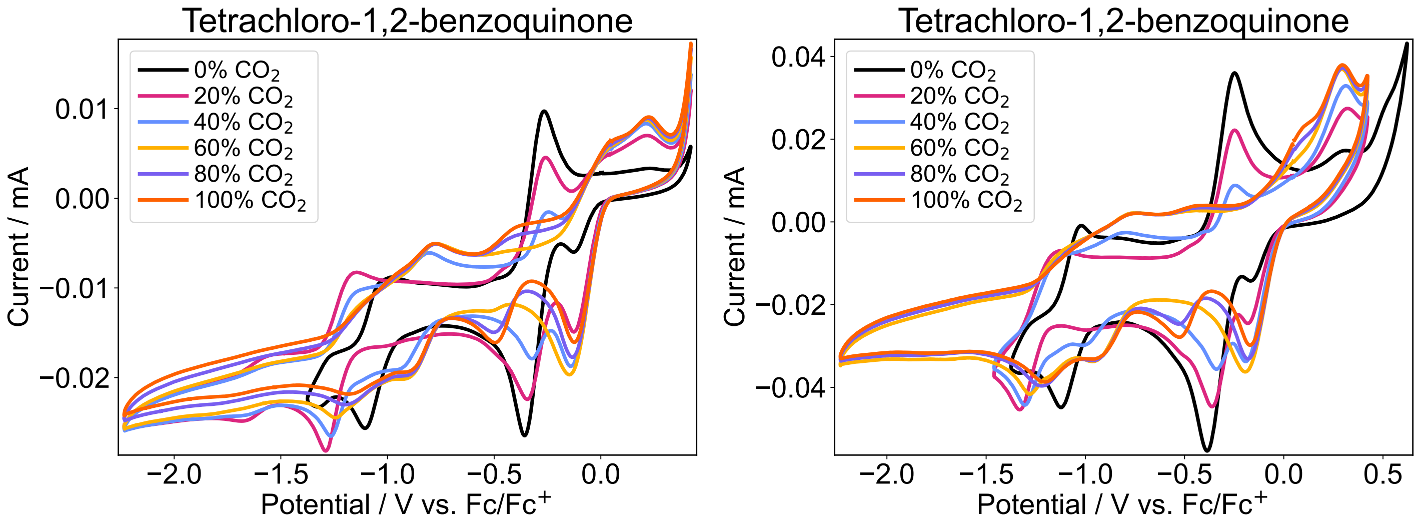

Figure S14: The cyclic voltammograms of tetrachloro-1,2-benzoquinone in DMSO with varying concentrations of CO_2. The left and right figures correspond to scan rates of 20 and 100 mV s^{-1} respectively.

For this quinone, unusual behaviour was observed. Firstly there appeared to be multiple reduction peaks, noticeably with a minor reduction peak under N_2 which becomes significantly more prominent when CO_2 is introduced. Secondly, it appears that the $Q^{\bullet -}/Q^{2-}$ reduction peak initially shifts in the negative direction when CO_2 is first introduced, and then shifts positively as the CO_2 concentration is progressively increased. This can not be explained by the model used for extracting binding constants for the other six quinones.

Once the cyclic voltammograms were complete, and after ferrocene was added to take an internal reference, it was noted that the solution had turned black. Initially the solution was a vibrant orange, and ferrocene is also orange in colour. This indicates that the quinone likely decomposed during the experiment.

For the above reasons, this quinone was not included in the calibration data set. The actual process of decomposition is possibly worth further investigation however as it is noted that other *ortho*-quinones are also unstable and from the computational work here it seems that *ortho*-quinones should in fact be the most promising candidated for electrochemical CO_2 capture, as long as stable forms can be found.

S6 Tabulated Results for Substituted Quinones

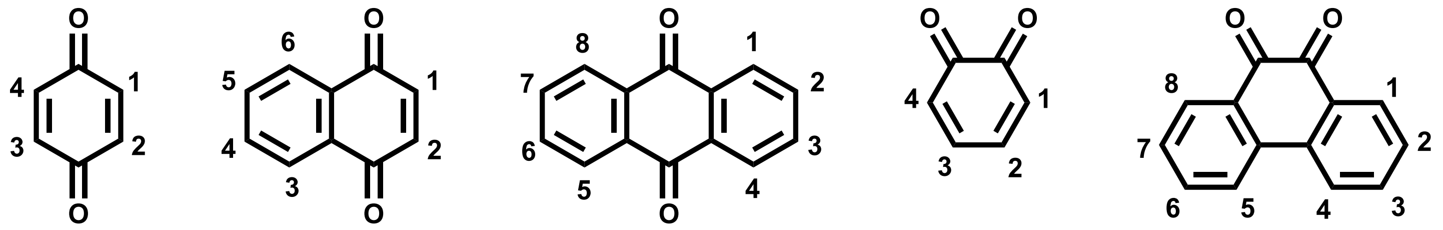

Figure S15: The structures and numbered positions of benzoquinone, naphthoquinone, anthraquinone, *ortho*-benzoquinone and phenanthenequinone from left to right respectively.

S6.1 Benzoquinones

Quinone	E_{2e} / V	log(K)	Quinone	E_{2e} / V	log(K)
BQ	-1.288	17.109	Br_2(1,2)	-1.016	9.503
F_2(1,2)	-1.036	10.530	Br_2(1,3)	-0.968	9.262
F_2(1,3)	-1.046	10.468	Br_2(1,4)	-0.969	8.415
F_2(1,4)	-1.029	9.795	Br_4(1,2,3,4)	-0.785	2.537
F_4(1,3)	-0.791	4.614	Me_2(1,2)	-1.457	18.202
Cl_2(1,2)	-1.022	9.464	Me_2(1,3)	-1.447	16.919
Cl_2(1,3)	-0.983	9.173	Me_2(1,4)	-1.444	15.724
Cl_2(1,4)	-0.984	9.858	Me_4(1,2,3,4)	-1.612	19.659
Cl_4(1,3)	-0.785	3.110			

Table S5: Table of the calculated and calibrated values of E_{2e} and log(K) for the halide and methyl substituted benzoquinones investigated here. All redox potentials are referenced against Fc^+/Fc.

S6.2 Naphthoquinones

Quinone	E_{2e} / V	log(K)	Quinone	E_{2e} / V	log(K)
NQ	-1.441	15.196	Cl_6(1,2,3,4,5,6)	-1.028	5.638
F_2(1,2)	-1.214	8.384	Br_2(1,2)	-1.169	6.468
F_2(3,6)	-1.372	14.560	Br_2(3,6)	-1.339	9.847
F_2(4,5)	-1.335	10.759	Br_2(4,5)	-1.285	9.489
F_4(1,2,3,6)	-1.139	8.145	Br_4(1,2,3,4)	-1.102	5.593
F_4(1,2,4,5)	-1.105	5.803	Br_4(1,2,4,5)	-1.026	2.272
F_4(3,4,5,6)	-1.274	11.054	Br_4(3,6,4,5)	-1.245	7.313
F_6	-1.017	4.655	Br_6(1,2,3,4,5,6)	-1.091	2.144
Cl_2(1,2)	-1.181	7.177	Me_2(1,2)	-1.575	15.401
Cl_2(3,6)	-1.368	13.870	Me_2(3,6)	-1.605	15.249
Cl_2(4,5)	-1.300	9.964	Me_2(4,5)	-1.504	13.046
Cl_4(1,2,3,6)	-1.123	8.537	Me_4(1,2,4,5)	-1.666	15.301
Cl_4(1,2,4,5)	-1.049	4.121	Me_4(3,6,4,5)	-1.674	15.116
Cl_4(3,4,5,6)	-1.268	11.067	Me_6	-1.806	16.295

Table S6: Table of the calculated and calibrated values of E_{2e} and log(K) for the halide and methyl substituted naphthoquinones investigated here. All redox potentials are referenced against Fc^+/Fc.

S6.3 Anthraquinones

Quinone	E_{2e} / V	log(K)	Quinone	E_{2e} / V	log(K)
AQ	-1.633	13.019	Br_2(1,5)	-1.571	11.866
F_2(1,5)	-1.550	11.994	Br_2(2,6)	-1.489	8.744
F_2(2,6)	-1.527	10.034	Br_4(1,4,5,8)	-1.551	7.412
F_4(1,4,5,8)	-1.451	9.689	Br_4(2,3,6,7)	-1.353	5.285
F_4(2,3,6,7)	-1.427	8.706	Br_6(1,2,4,5,6,8)	-1.445	2.885
F_6(1,2,4,5,6,8)	-1.333	7.394	Br_8	-1.372	0.351
F_8	-1.268	6.243	Me_2(1,5)	-1.789	16.090
Cl_2(1,5)	-1.542	12.712	Me_2(2,6)	-1.690	12.569
Cl_2(2,6)	-1.477	7.775	Me_4(1,4,5,8)	-1.971	16.132
Cl_4(1,4,5,8)	-1.525	8.907	Me_4(2,3,6,7)	-1.757	12.900
Cl_4(2,3,6,7)	-1.353	5.790	Me_6(1,2,4,5,6,8)	-1.999	13.964
Cl_6(1,2,4,5,6,8)	-1.451	8.186	Me_8	-2.070	13.035
Cl_8	-1.385	3.565			

Table S7: Table of the calculated and calibrated values of E_{2e} and log(K) for the halide and methyl substituted anthraquinones investigated here. All redox potentials are referenced against Fc^+/Fc.

S6.4 *ortho*-Benzoquinones

Quinone	E_{2e} / V	log(K)	Quinone	E_{2e} / V	log(K)
oBQ	-1.157	14.407	Cl_4(1,2,3,4)	-0.680	2.767
F_2(1,4)	-0.876	8.989	Br_2(1,4)	-0.824	5.973
F_2(2,3)	-1.042	10.906	Br_2(2,3)	-0.894	7.837
F_4	-0.752	7.035	Br_4	-0.688	1.479
Cl_2(1,2)	-0.914	6.833	Me_2(1,4)	-1.307	18.559
Cl_2(1,3)	-0.882	9.944	Me_2(2,3)	-1.318	17.261
Cl_2(1,4)	-0.836	6.755	Me_4	-1.476	19.555
Cl_2(2,3)	-0.918	9.391			

Table S8: Table of the calculated and calibrated values of E_{2e} and log(K) for the halide and methyl substituted *ortho*-benzoquinones investigated here. All redox potentials are referenced against Fc^+/Fc.

S6.5 Phenanthrenequinones

Quinone	E_{2e} / V	log(K)	Quinone	E_{2e} / V	log(K)
PAQ	-1.428	13.568	Br_2(1,8)	-1.454	11.099
F_2(1,8)	-1.405	12.773	Br_2(2,7)	-1.282	10.000
F_2(2,7)	-1.286	10.309	Br_2(3,6)	-1.270	9.114
F_2(3,6)	-1.372	11.111	Br_2(4,5)	-1.373	10.516
F_2(4,5)	-1.328	10.995	Br_4(1,8,2,7)	-1.342	8.313
F_4(1,8,2,7)	-1.281	10.156	Br_4(1,8,3,6)	-1.316	8.057
F_4(1,8,3,6)	-1.345	10.156	Br_4(1,8,4,5)	-1.421	10.460
F_4(1,8,4,5)	-1.299	9.840	Br_4(2,7,3,6)	-1.157	6.039
F_4(2,7,3,6)	-1.239	9.329	Br_4(2,7,4,5)	-1.236	6.005
F_4(2,7,4,5)	-1.188	8.189	Br_4(3,6,4,5)	-1.275	8.148
F_4(3,6,4,5)	-1.275	10.510	Br_6(1,8,2,7,3,6)	-1.242	6.221
F_6(1,8,2,7,3,6)	-1.220	8.717	Br_6(1,8,2,7,4,5)	-1.288	5.796
F_6(1,8,2,7,4,5)	-1.172	6.945	Br_6(2,7,3,6,4,5)	-1.120	4.428
F_6(2,7,3,6,4,5)	-1.138	7.111	Br_8	-1.173	2.133
F_8	-1.114	5.665	Me_2(1,8)	-1.632	15.855
Cl_2(1,8)	-1.435	11.430	Me_2(2,7)	-1.491	13.020
Cl_2(2,7)	-1.263	9.804	Me_2(3,6)	-1.522	15.103
Cl_2(3,6)	-1.300	9.908	Me_2(4,5)	-1.556	14.325
Cl_2(4,5)	-1.340	10.704	Me_4(1,8,2,7)	-1.671	13.544
Cl_4(1,8,2,7)	-1.313	7.963	Me_4(1,8,3,6)	-1.714	16.095
Cl_4(1,8,3,6)	-1.351	9.261	Me_4(1,8,4,5)	-1.801	18.408
Cl_4(1,8,4,5)	-1.361	9.241	Me_4(2,7,3,6)	-1.577	13.950
Cl_4(2,7,3,6)	-1.204	8.113	Me_4(2,7,4,5)	-1.585	13.332
Cl_4(2,7,4,5)	-1.207	6.927	Me_4(3,6,4,5)	-1.628	14.250
Cl_4(3,6,4,5)	-1.230	7.233	Me_6(1,8,2,7,3,6)	-1.765	16.649
Cl_6(1,8,2,7,3,6)	-1.259	7.575	Me_6(1,8,2,7,4,5)	-1.869	16.688
Cl_6(1,8,2,7,4,5)	-1.274	7.525	Me_6(2,7,3,6,4,5)	-1.660	14.714
Cl_6(2,7,3,6,4,5)	-1.120	4.855	Me_8	-1.903	17.322
Cl_8	-1.203	7.488			

Table S9: Table of the calculated and calibrated values of E_{2e} and log(K) for the halide and methyl substituted phenanthrenequinones investigated here. All redox potentials are referenced against Fc^+/Fc.

S7 E_{2e} to Avoid Oxygen Reduction

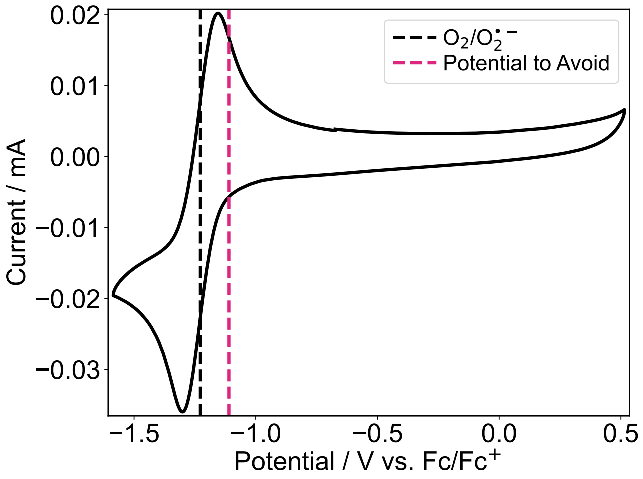

Figure S16: Cyclic voltammogram at 50 mVs^{-1} of DMSO with 0.1M $TBAPF_6$ after purging with O_2. The $O_2/O_2^{\bullet-}$ redox potential is labelled (black dashed line) as well as the potential at which 1% of O_2 would be expected to be reduced (pink dashed line).

The $O_2/O_2^{\bullet-}$ redox potential was found to be -1.23 V vs Fc/Fc^+. We then calculated the potential at which 1% of present O_2 would be reduced, using the Nernst equation, Eq. (S18).

$$E_{1\%\ \mathrm{O_2\ Reduction}} = E^{\ominus} - \frac{RT}{F}\ln\left(\frac{1}{99}\right) \tag{S18}$$

At $T = 25°\mathrm{C}$, it is calculated that $E_{1\%\ \mathrm{O_2\ Reduction}} = -1.11\mathrm{V}$. To minimise the possibility of O_2 being able to oxidise quinone dianions back to the radical anion, the $Q^{\bullet-}/Q^{2-}$ potential should be positive of this value.

To calculate how positive E_{2e} needs to be in order for the $Q^{\bullet-}/Q^{2-}$ potential to be positive of $E_{1\%\ \mathrm{O_2\ Reduction}}$, the separation between $Q^{\bullet-}/Q^{2-}$ and $Q/Q^{\bullet-}$ must be considered. This separation is tabulated for the six quinones investigated earlier and a mean value taken with the error calculated as the standard deviation, Table S10.

Quinone	Redox Potentials Separation / V
Tetrachloro-1,4-benzoquinone	0.821
Octafluoroanthraquinone	0.627
1,4-Difluoroanthraquinone	0.684
1-Chloroanthraquinone	0.673
Anthraquinone	0.700
Naphthoquinone	0.786
Average Separation	0.72 ± 0.07

Table S10: Experimental values for the separation between $Q^{\bullet-}/Q^{2-}$ and $Q/Q^{\bullet-}$ for six various quinones. The mean separation is calculated and the error is stated as the standard deviation of the results.

Using the found value for average separation of the two redox potentials, the two-electron redox potential at which $Q^{\bullet-}/Q^{2-}$ is positive of $E_{1\%\ \mathrm{O_2\ Reduction}}$ can be calculated, Eq. (S19).

$$E_{2e}^{\mathrm{Avoid\ O_2\ Reduction}} = E_{1\%\ \mathrm{O_2\ Reduction}} + \frac{\mathrm{Average\ Separation}}{2} = -0.75\ \mathrm{V} \tag{S19}$$

S8 Considering Asymmetric Quinones

In this work the presented trade-offs correspond to symmetrically substituted quinones. This methodology was chosen to limit the required number of structures to be generated to a reasonable level, reducing the required computational cost. However it is possible that this restriction on the explored chemical space may have excluded the most promising species from this study. As a compromise, all of the asymmetric fluorine-substituted naphthoquinones were investigated using the same computational methods, however in this case for each structure the binding constant of CO_2 to each oxygen independently had to be considered, as they are no longer equivalent.

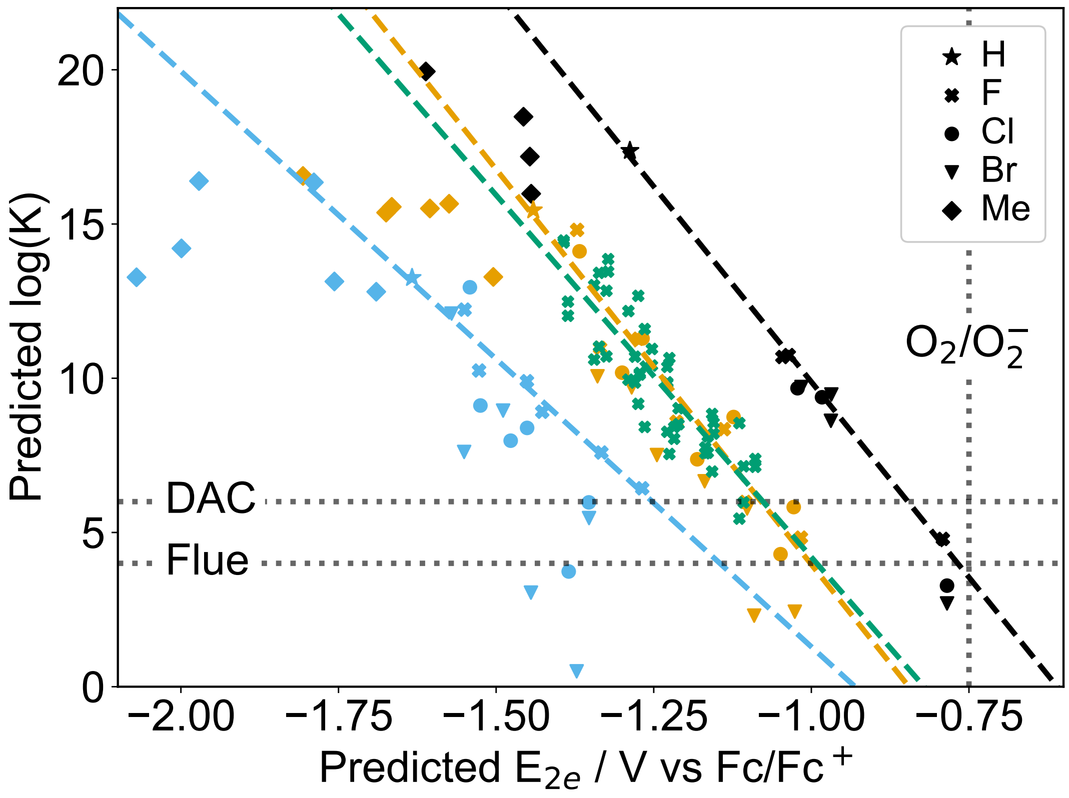

Figure S17: The predicted binding constants of various quinones versus their calculated two-electron redox potentials. In black, yellow, and blue are symmetrically substituted benzoquinones, naphthoquinones and anthraquinones respectively. In green are asymmetrically fluorine-substituted naphthoquinones. For each of the quinone sets there is a line-of-best-fit corresponding only to the fluorine substituted quinones.

When the asymmetric naphthoquinones are overlaid on the symmetric results(Fig. S17, green crosses) and a new line of best fit calculated (Fig. S17, green dashed line), we see that there is no significant difference to the trade-off line previously calculated for naphthoquinone.

From these results the authors believe that they were justified in only considering the symmetrically-substituted quinones to obtain the general trade-off line for each quinone. The authors would then suggest that if a promising quinone is found that the full chemical space for that one quinone should be explored.

S8.1 Tabulated Results for Asymmetrically Substituted Naphthoquinones

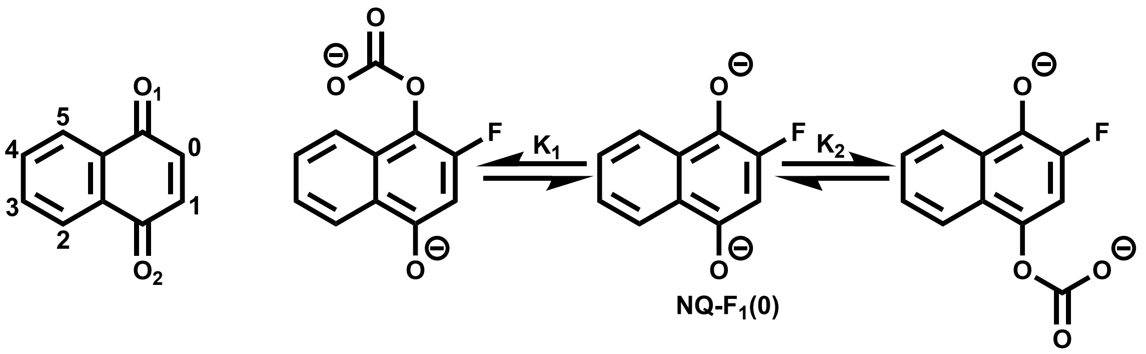

Figure S18: Numbering convention for naphthoquinone (left most) and an example for NQ$-F_1(0)$ showing which binding constant refers to CO_2 binding on which oxygen atom.

Quinone	E_{2e} / V	$\log(K_1)$	$\log(K_2)$	Quinone	E_{2e} / V	$\log(K_1)$	$\log(K_2)$
$F_1(0)$	-1.325	12.834	10.707	$F_3(0,2,4)$	-1.211	8.510	9.028
$F_1(2)$	-1.393	14.399	14.465	$F_3(0,2,5)$	-1.253	10.949	10.412
$F_1(3)$	-1.386	12.484	12.025	$F_3(0,3,4)$	-1.218	8.415	8.038
$F_2(0,2)$	-1.272	10.170	10.119	$F_3(0,3,5)$	-1.229	9.831	8.264
$F_2(0,3)$	-1.275	12.671	9.170	$F_3(0,4,5)$	-1.225	10.652	7.545
$F_2(0,4)$	-1.264	11.596	8.421	$F_3(2,3,4)$	-1.290	9.953	12.177
$F_2(0,5)$	-1.280	10.699	9.860	$F_3(2,3,5)$	-1.322	13.861	13.456
$F_2(2,3)$	-1.344	10.606	13.017	$F_4(0,1,2,3)$	-1.114	5.449	8.544
$F_2(2,4)$	-1.337	11.024	13.416	$F_4(0,1,2,4)$	-1.107	5.976	7.145
$F_3(0,1,2)$	-1.164	7.586	8.114	$F_4(0,2,3,4)$	-1.168	7.568	7.756
$F_3(0,1,3)$	-1.157	8.841	6.974	$F_5(0,1,2,3,5)$	-1.089	7.382	7.128
$F_3(0,2,3)$	-1.228	9.868	10.369	$F_5(0,2,3,4,5)$	-1.155	8.561	8.204

Table S11: Tabulated results for the calculated two-electron redox potentials, and two binding constants for all the asymmetrically fluorine substituted naphthoquinones. All redox potentials are referenced against Fc^+/Fc. The numbering convention for the substitutions is given in Fig. S18, as well as the convention for which CO_2 capture each binding constant refers to.

S9 Calculating Aromatic Stabilisation Energy

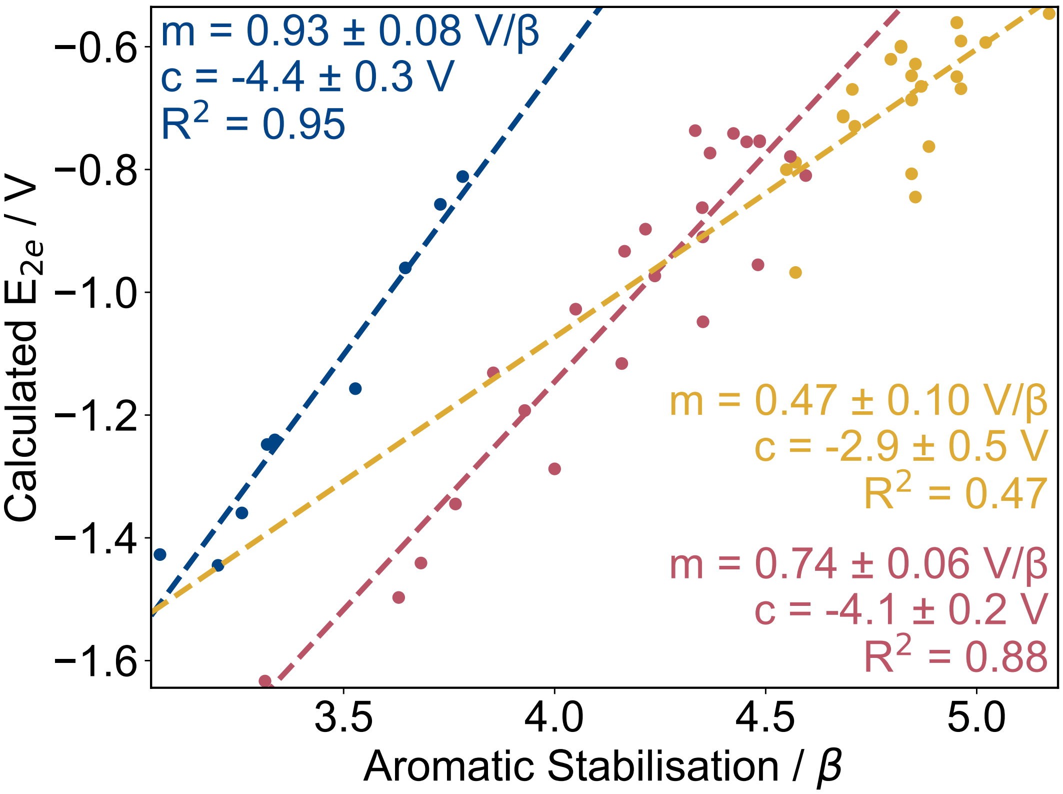

Figure S19: E_{2e} calculated using the described computational methods and calibrated using the experimental results, plotted against the increase in aromatic stabilisation energy calculated from Hückel theory. The data points for *ortho* quinones are plotted in blue, the yellow points correspond to the quinones where the structure cannot be drawn without the use of radicals, and the red points correspond to all the other quinones considered. For the three distinct data sets, linear regression is performed from which the gradients, y-intercept, associated errors, and R^2 are yielded.

S9.1 Calculating β

The value of β can be found in kJ mol^{-1} by utilising the following equation which relates Gibbs energy to the standard redox potential, Eq. (S20).

$$\Delta G = -nFE^{\ominus}_{2e} \tag{S20}$$

In this case we calculated the two-electron redox-potential, therefore $n = 2$. The value of β in kJ mol^{-1} is therefore obtained by multiplying the value of the gradient by $-2F/1000$ where F (the Faraday constant) is in units of s A mol^{-1}. The errors for β presented in Table S12 are also calculated by multiplying the gradient errors in Fig. S19 by $-2F/1000$.

Quinone Set	Gradient / Vβ^{-1}	β / kJ mol^{-1}
Ortho	0.93 ± 0.08	-180 ± 15
Non-*Ortho*	0.74 ± 0.06	-143 ± 11
Non-Kekulé	0.47 + 0.10	-91 ± 19

Table S12: The calculated values of β from the linear regression plots in Fig. S19.

S9.2 Tabulated Results for Aromatic Stabilisation Study

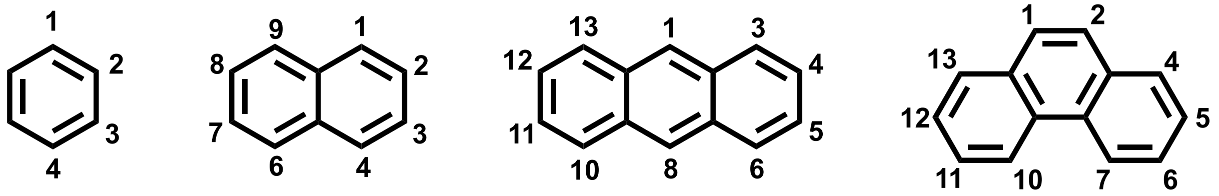

Figure S20: Numbered sites on benzene, naphthacene, anthracene and phenanthracene.

Quinone	χ / β	E_{2e} / V	log(K) 1	log(K) 2	Quinone	χ / β	E_{2e} / V	log(K) 1	log(K) 2
BQ-1,2	3.528	-1.157	14.322		PAQ-1,12	4.424	-0.741	4.678	2.425
BQ-1,3	5.172	-0.546	9.993		PAQ-1,13	4.846	-0.807	7.103	5.779
BQ-1,4	4.000	-1.288	17.954		PAQ-1,4	4.050	-1.028	4.982	4.813
NQ-1,2	3.259	-1.359	13.446	14.557	PAQ-1,5	4.797	-0.620	4.864	4.167
NQ-1,3	4.963	-0.668	7.463	7.659	PAQ-1,6	4.159	-1.116	7.993	7.237
NQ-1,4	3.683	-1.441	15.196		PAQ-1,7	4.705	-0.670	3.712	4.355
NQ-1,6	4.166	-0.933	6.920		PAQ-1,10	4.368	-0.773	1.552	2.696
NQ-1,7	4.855	-0.628	5.849	4.650	PAQ-4,11	4.455	-0.755	3.423	4.129
NQ-1,8	4.237	-0.973	6.210	9.819	PAQ-4,12	4.821	-0.601	3.264	2.578
NQ-1,9	4.855	-0.845	8.593		PAQ-4,13	4.333	-0.737	2.656	
NQ-2,3	3.729	-0.857	8.560		PAQ-4,5	3.319	-1.248	10.690	11.223
NQ-2,7	4.352	-1.048	9.820		PAQ-4,6	4.953	-0.649	6.662	5.785
NQ-2,8	4.963	-0.591	7.014		PAQ-4,7	3.765	-1.345	11.128	9.875
AQ-1,3	4.571	-0.968	3.996	6.201	PAQ-4,10	4.684	-0.712	2.843	2.166
AQ-1,4	3.929	-1.193	4.627	7.487	PAQ-5,11	4.953	-0.560	3.620	3.428
AQ-1,5	4.571	-0.788	4.819	1.104	PAQ-5,12	4.595	-0.810	4.667	
AQ-1,6	3.855	-1.131	3.161	5.664	PAQ-5,13	4.821	-0.599	3.188	2.493
AQ-1,8	3.314	-1.633	13.019		PAQ-5,6	3.646	-0.960	9.614	9.986
AQ-3,11	4.711	-0.730	3.118	1.834	PAQ-5,7	5.021	-0.593	6.981	5.319
AQ-3,12	4.351	-0.910	5.318	3.001	PAQ-5,10	4.486	-0.753	4.831	3.125
AQ-3,13	4.549	-0.800	2.538		PAQ-6,11	4.559	-0.779	4.218	
AQ-3,4	3.202	-1.445	12.672	16.112	PAQ-6,12	4.953	-0.561	3.385	3.423
AQ-3,5	4.887	-0.763	4.478	5.690	PAQ-6,7	3.337	-1.240	10.816	10.538
AQ-3,6	3.630	-1.497	11.842		PAQ-6,10	4.846	-0.686	5.271	1.005
AQ-3,10	4.215	-0.897	2.302		PAQ-7,11	4.846	-0.687	1.070	3.800
AQ-4,11	4.482	-0.955	6.225		PAQ-7,12	4.486	-0.755	5.037	3.186
AQ-4,12	4.868	-0.665	4.479		PAQ-7,13	4.684	-0.715	2.199	4.019
AQ-4,5	3.782	-0.812	5.644		PAQ-7,10	4.350	-0.862	6.083	
PAQ-1,2	3.065	-1.427	13.520						
PAQ-1,11	4.846	-0.647	2.386	3.212					

Table S13: Calculated aromatic stabilisation, χ, two-electron redox potential and log(K) values for all possible benzoquinones (BQ), naphthoquinones (NQ), anthraquinones (AQ) and phenanthrenequinones (PAQ). All redox potentials are referenced against Fc^{+}/Fc. The names of *ortho* quinones, non-*ortho* quinones, and non-Kekulé quinones are coloured blue, red and yellow respectively. The carbonyl position labels correspond to the positions shown in Fig. S20

S9.3 Relationship Between CO_2 Binding Constant and Aromatic Stabilisation

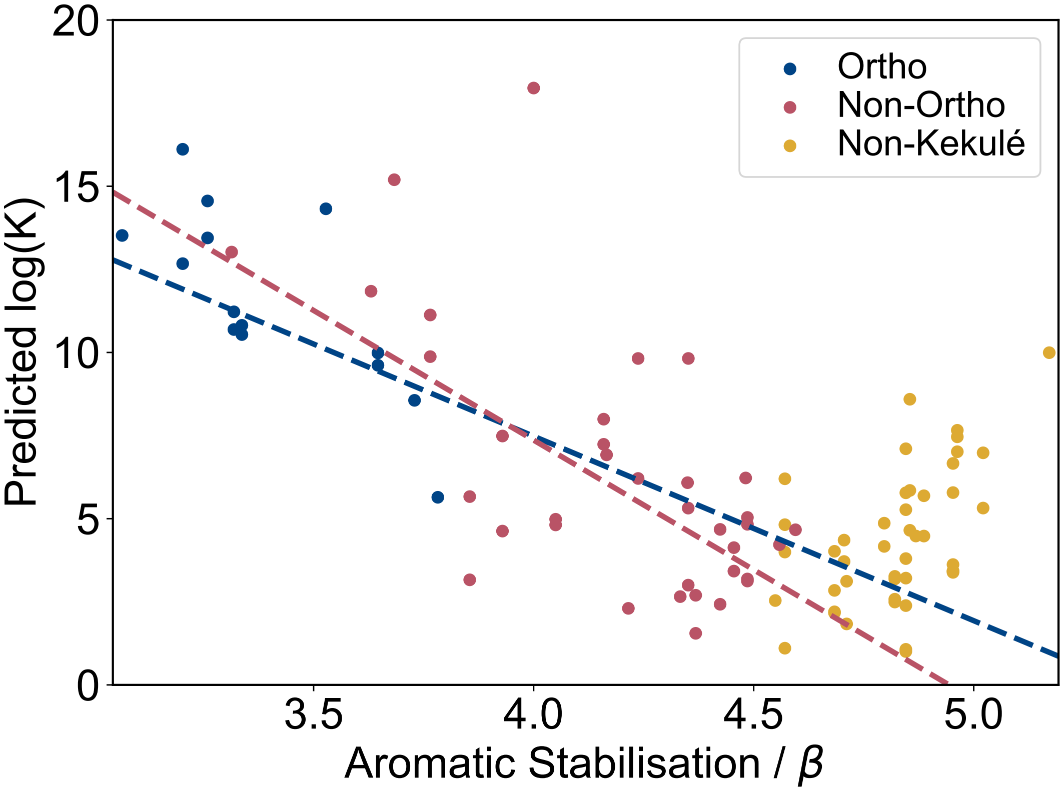

Figure S21: $\log(K)$ calculated using the described computational methods and calibrated using the experimental results, plotted against the increase in aromatic stabilisation energy calculated from Hückel theory. The data points for ortho quinones are plotted in blue, the yellow points correspond to the quinones where the structure cannot be drawn without the use of radicals, and the red points correspond to all the other quinones considered. Lines of best fir are provided for the *ortho* and non-*ortho* data sets.

S10 2,3-Naphthoquinone

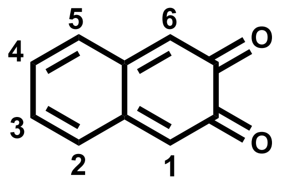

Figure S22: 2,3-Naphthoquinone with labelled positions.

S10.1 Tabulated Results for Fluorinated 2,3-NQ

Quinone	E_{2e} / V	log(K) 1	log(K) 2	Quinone	E_{2e} / V	log(K) 1	log(K) 2
2,3-NQ	-0.855	8.361		F_3(3,4,6)	-0.655	4.322	4.426
F_1(1)	-0.742	5.808	5.722	F_3(3,5,6)	-0.609	3.021	5.086
F_1(3)	-0.811	7.045	8.130	F_4(1,2,3,6)	-0.508	0.899	1.498
F_1(5)	-0.758	7.203	7.094	F_4(1,2,4,5)	-0.521	2.190	1.839
F_2(1,2)	-0.659	4.926	4.356	F_4(1,2,5,6)	-0.469	0.848	
F_2(1,6)	-0.637	3.057		F_4(1,3,4,5)	-0.561	1.952	2.980
F_2(2,3)	-0.717	5.998	7.189	F_4(1,3,4,6)	-0.542	1.573	
F_2(2,4)	-0.715	7.075	4.734	F_4(1,3,5,6)	-0.501	0.341	1.740
F_2(2,5)	-0.667	4.713		F_4(2,3,4,5)	-0.583	3.373	
F_2(2,6)	-0.648	4.362	3.319	F_4(2,3,4,6)	-0.561	3.022	1.818
F_2(3,4)	-0.759	6.991		F_4(2,4,5,6)	-0.520	2.964	1.131
F_2(3,5)	-0.717	6.133	5.873	F_4(3,4,5,6)	-0.567	3.030	4.078
F_2(3,6)	-0.694	4.397	5.561	F_5(1,2,3,4,5)	-0.477	1.032	1.732
F_2(4,6)	-0.698	5.501	4.392	F_5(1,2,4,5,6)	-0.424	0.546	-0.555
F_3(1,2,3)	-0.615	3.965	4.184	F_5(1,3,4,5,6)	-0.457	0.177	0.619
F_3(1,2,5)	-0.566	2.395	3.057	F_6(1,2,3,4,5,6)	-0.377	-0.518	
F_3(1,3,5)	-0.604	2.078	4.125				
F_3(1,3,6)	-0.586	1.795	2.760				
F_3(1,5,6)	-0.552	1.842	2.136				
F_3(2,3,4)	-0.679	5.929	4.685				
F_3(2,3,6)	-0.602	3.202	3.093				
F_3(2,4,5)	-0.622	4.457	3.574				
F_3(2,4,6)	-0.602	4.133	1.943				
F_3(2,5,6)	-0.565	3.097	2.332				

Table S14: Tabulated results of fluroine substituted 2,3-naphthoquinones. Quinones are labelled by number of fluorine substitutions and their respective positions corresponding to the labelled positions in Fig. S22. All redox potentials are referenced against Fc^+/Fc. Quinones predicted to have at least one data point in the flue gas region are coloured orange, and those with at least one data point in the DAC region are coloured green, all others are black.

S10.2 Attempt to Synthesise 2,3-Naphthoquinone

It was first attempted to synthesise the oxidised form of 2,3-naphthoquinone from the readily available hydroquinone. The quinone has reportedly been synthesised previously using the same starting material and oxidising agent,[17] however no yields or spectroscopic data were provided. A similar approach has also been used in the synthesis of *ortho*-benzoquinone starting from the the corresponding hydroquinone.[18] In this attempted synthesis, the conditions from the *ortho*-benzoquinone synthesis were used.

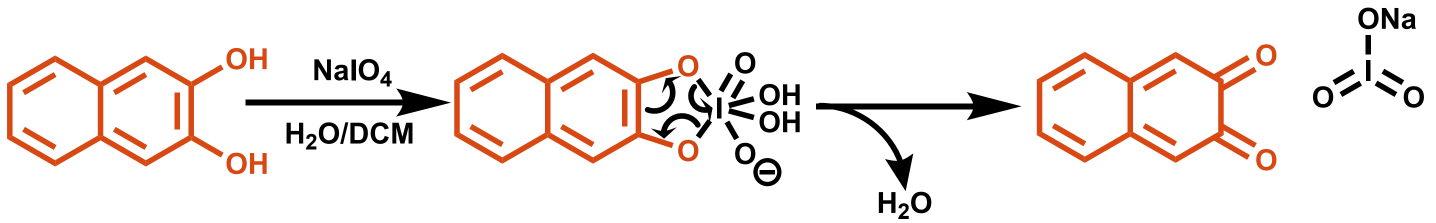

Figure S23: Attempted synthesis of 2,3-naphthoquinone from the hydroquinone using sodium periodate in water as the oxidising agent.

Sodium periodate (400 mg, 1.87 mmol) was dissolved in deionised water (10 mL) and cooled to 0°C under N_2. 2,3-Dihydroxynapthalene (pale pink) was suspended in DCM (10 mL) under N_2 at 0°C. The sodium periodate solution was added slowly to the DCM suspension with vigorous stirring under N_2 at 0°C. Immediately a vibrant yellow colour emerged. The mixture was further stirred under the same conditions for ten minutes. The suspension was filtered *in vacuo* and washed with deionised water to obtain a brown precipitate (0.165 g). The precipitate was then dried *in vacuo* on the Schlenk line at 0°C for two hours to yield the product (0.132 g) as a brown and flaky solid. A ^{1}H-NMR spectra of the product was taken in D_6−DMSO, as well as of the hydroquinone, Fig. S24.

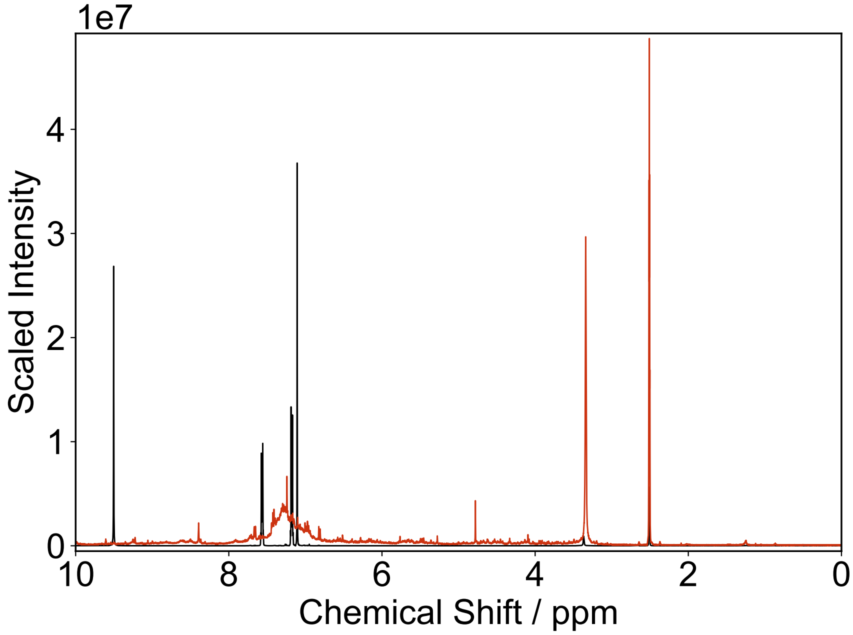

Figure S24: Black: ^{1}H NMR spectra of 2,3-dihydroxynapthalene in D_6−DMSO. Orange: ^{1}H NMR spectra of dried product from 2,3-dihydroxynapthalene oxidation, in D_6−DMSO.

NMR indicates that a clean product was not obtained. There appears to be a broad range of signals in the aromatic region, possibly indicating some type of polymerisation, or decomposition of the product. It has previously been reported that *ortho*-benzoquinone is unstable.

S10.3 Synthesis of 2,3-Naphthoquinone Dianion

Napthalene-2,3-diol (250 mg, 1.56 mmol) was dissolved in THF (anhydrous, 20 mL) and kept under N_2. Sodium hydride (60% dispersion in mineral oil, 128 mg, 3.20 mmol, 2.05 eq) was suspended in THF (anhydrous, 20 mL) in a three-neck round-bottom flask with a stirrer bar and condenser attached. The sodium hydride suspension was put under N_2 and cooled to 0°C via an ice-bath. The diol solution was added slowly to the sodium hydride suspension at 0°C with stirring. The mixture immediately turned a cloudy yellow and stirring was allowed to continue for a further ten minutes. Methanol (1 mL) was then added to quench remaining sodium hydride. The precipitate was isolated via filtration *in vacuo* to yield the crude product as a pale pink solid (105 mg). The crude product was dried in a vacuum oven at 90°C for one week to then yield the product, sodium napthalene-2,3-bis(olate) as a brown solid (70 mg, 0.34 mmol, 22%).

The bis(olate) NMR sample was prepared from dissolving 2 mg of the product in 0.7 mL of D_6−DMSO, the solution appeared a greyish blue. The bis(carbonate) NMR sample was prepared by dissolving 2 mL of the bis(olate) in 0.7 mL. of D_6−DMSO and then briefly bubbling CO_2 through the solution, which turned a clear dark blue.

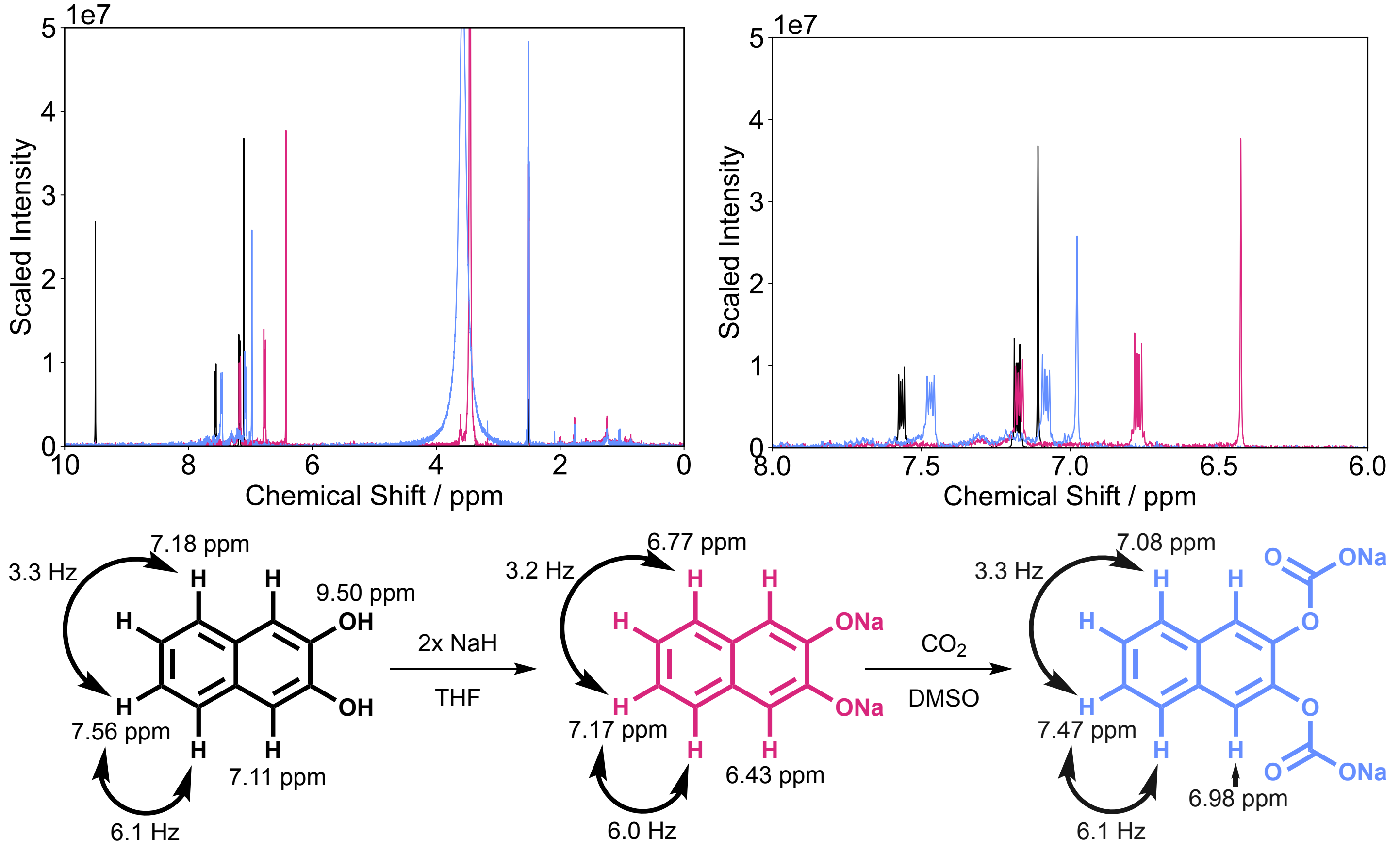

Figure S25: ^{1}H NMR 400 MHz of napthalene-2,3-diol, sodium napthalene-2,3-bis(olate) (red), and sodium napthalene-2,3-diyl bis(carbonate) (blue) in D_6−DMSO. The structures and assignments of each of the three molecules are also presented.

S10.4 Electrochemistry

After running all of the cyclic voltammograms on the 2,3-napthoquinone dianion electrolyte solution (5 mM in DMSO with 0.1 M $TBAPF_6$ counter electolyte), ferrocene was added so that an internal reference could be taken, as was standard procedure with all the previous cyclic voltammogram experiments conducted in this study. It was observed that the ferrocene wave, Fig. S26 right, was unusually low current and ill-defined, especially when compared to previous voltammograms where ferrocene has been used as internal reference, Fig. S26 left.

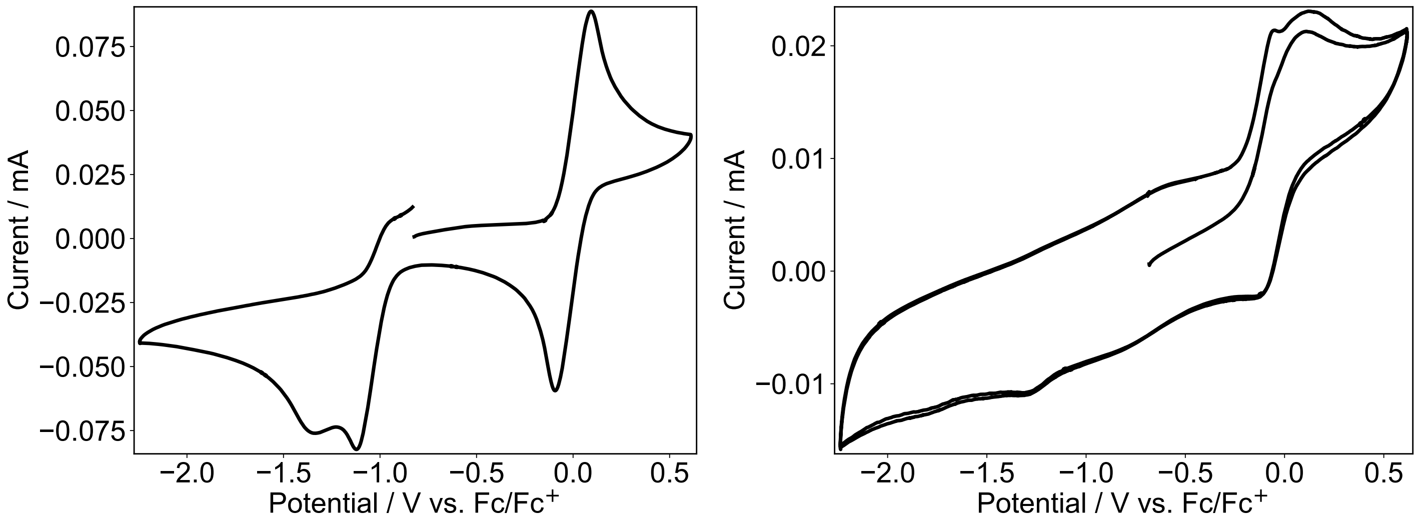

Figure S26: Left: Cyclic voltammogram of 5 mM napthoquinone solution in DMSO with 0.1M $TBAPF_6$ supporting electrolyte after addition of ferrocene, ran under CO_2 atmosphere at 100 mVs^{-1}. Right: Cyclic voltammogram of 5 mM sodium napthalene-2,3-bis(olate) solution in DMSO with 0.1M $TBAPF_6$ supporting electrolyte after addition of ferrocene, ran under CO_2 atmosphere at 100 mVs^{-1}.

During cleaning of the setup it appeared that there was a slick substance on the glassy working electrode, which had been polished prior to running the voltammetry experiments. It was hypothesised that this residue led to the weak and poorly-defined ferrocene wave observed. It was further hypothesised that this residue was due to decomposition of 2,3-naphthoquinone generated *in-situ* at the working electrode during operation. This is further evidence that the oxidised form of 2,3-naphthoquinone is unstable and is likely the reason that good electrochemistry could not be obtained for this molecule.

S11 Dichloro-2,3-Naphthoquinone

S11.1 Chlorination of Napthalene-2,3-diol

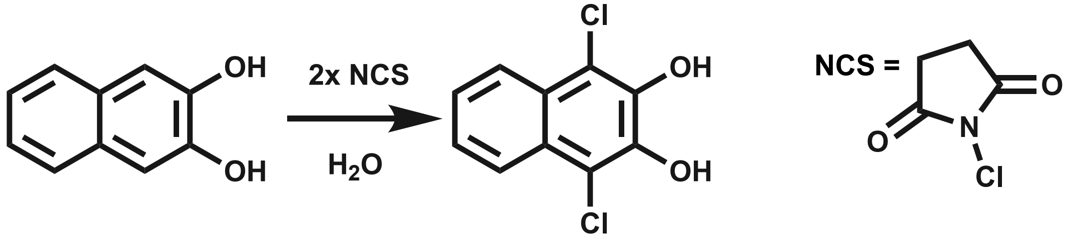

Napthalene-2,3-diol (104 mg, 0.649 mmol) was first suspended in deionised water (5 mL) with stirring. N-chlorosuccinimide (173 mg, 1.296 mmol, 2.00 equiv) was then added to the stirring suspension which immediately began to turn yellow. The suspension was then allowed to stir under N_2 for 2 hours. The precipitate was isolated via Buchner filtration and washed with deionised water. The yellow precipitate (122 mg) was collected and dried *in vacuo* overnight at 90°C, to then yield 94 mg of dried product. 10 mg of the dried product was dissolved in 1 mL of D_6−DMSO for NMR analysis.

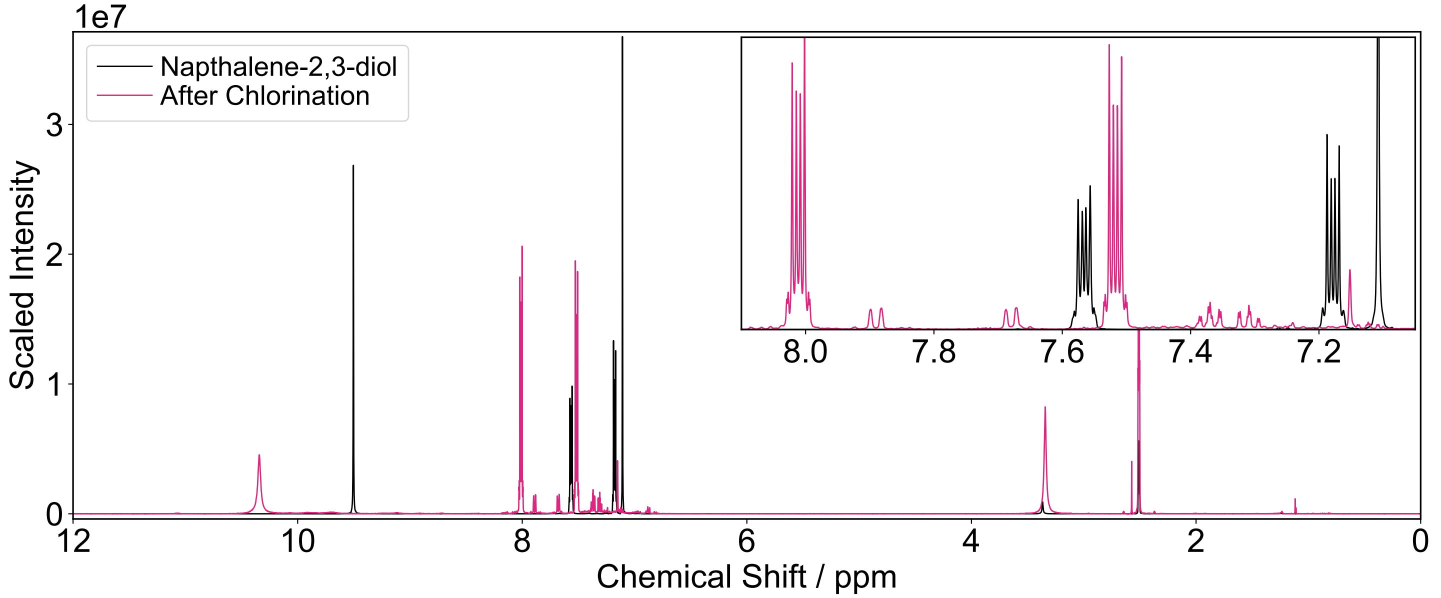

The NMR reveals that the major product is the dichlorinated hydroquinone, with chlorination in the *ortho* positions to the carbonyls (determined through multiplicity pattern). A minor product which likely corresponds to the mono-chlorinated product is also present as approximately 7% of the total product (determined through integration).

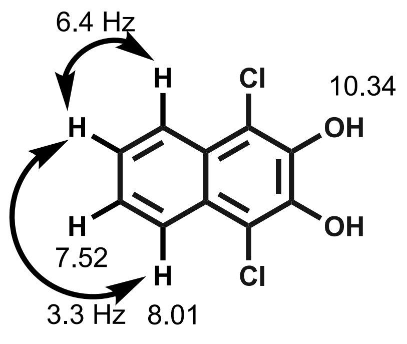

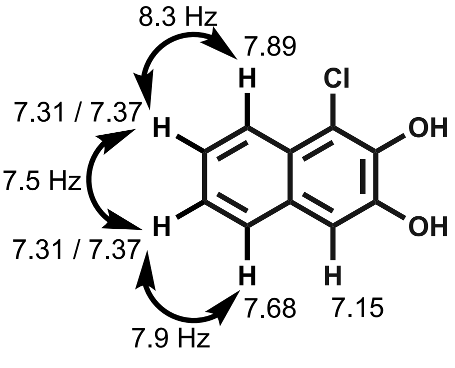

S11.2 Deprotonation of 1,4-Dichloronapthalene-2,3-diol

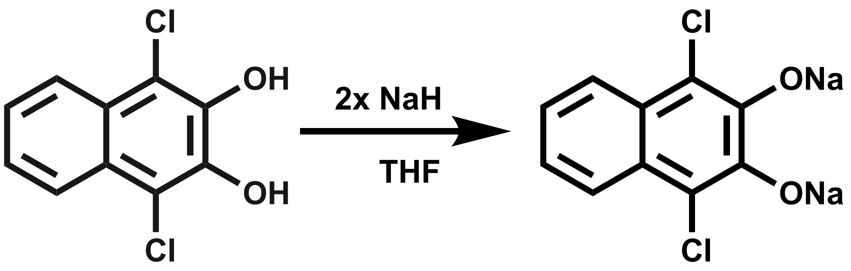

The mono-dichloro mixed product (66 mg, 0.291 mmol) was dissolved in anhydrous THF (5 mL) to form a golden orange solution. NaH (60% in mineral oil, 23 mg, 0.575 mmol, 2.0 eq) was suspended in anhydrous THF (4 mL) at 0°C under N_2 with stirring. The hydroquinone solution was then added slowly and the solution immediately took on a dark orange colour. The solution was allowed to stir under N_2 for a further two hours. The solvent was then removed *in vacuo* and the isolated product dried in a vacuum oven at 90°C overnight to yield the product (28 mg) as a black solid. 7 mg was dissolved in 1 mL of D_6−DMSO for analysis via NMR.

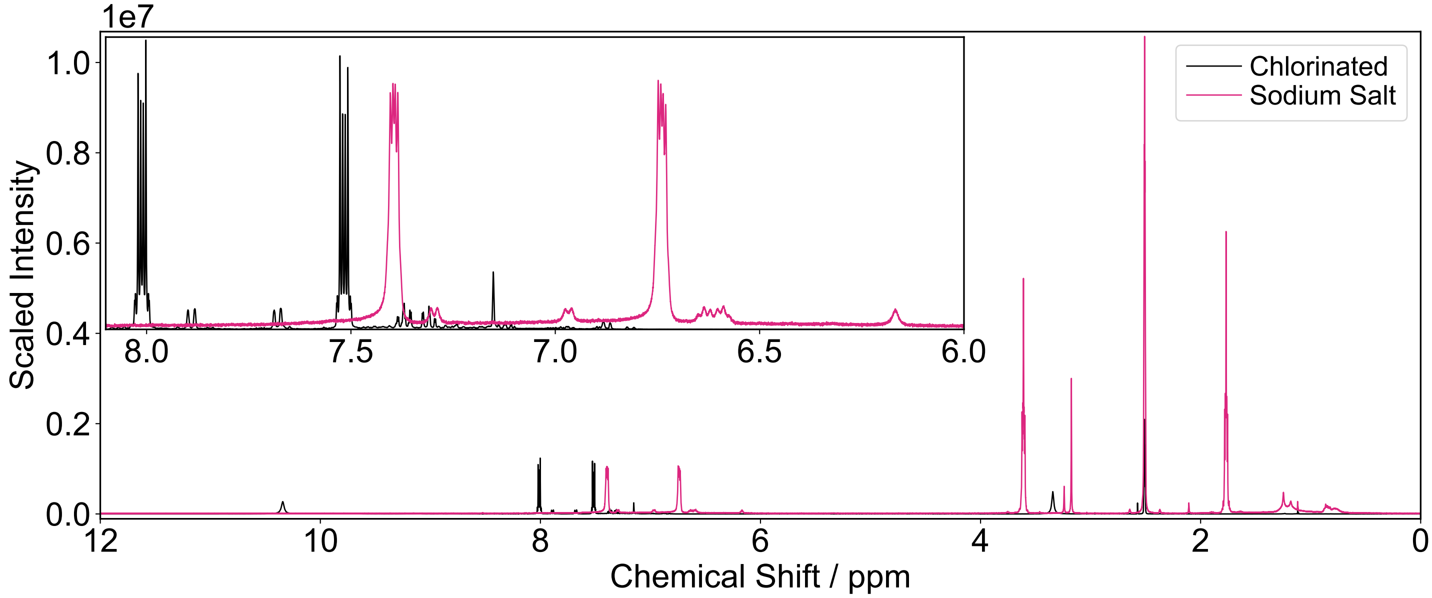

Looking at the aromatic region at 6.0-8.1 ppm, we see that both the monochlo and dichloro hydroquinones have become more shielded, which agrees with forming the dianion. Also the alcohol signal at 10.34ppm has been lost, again supporting formation of the dianion.

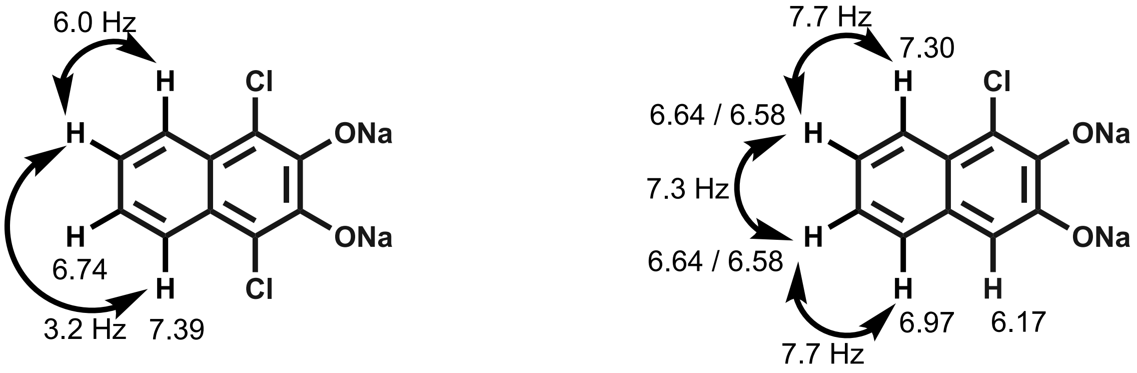

S11.3 Reaction of Sodium Salt with CO_2

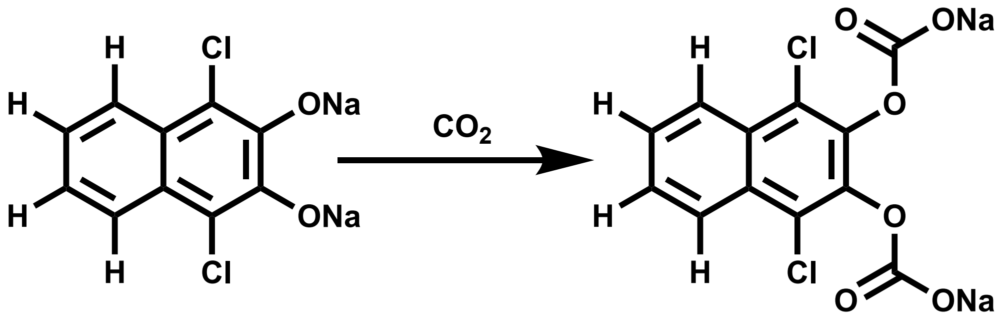

The NMR sample prepared with 7 mg of the sodium salt product was then purged with CO_2 (25 mL min^{-1}, 5 mins) with swirling. The D_6−DMSO solution changed from a golden to a green colour. NMR analysis was then ran again on this sample.

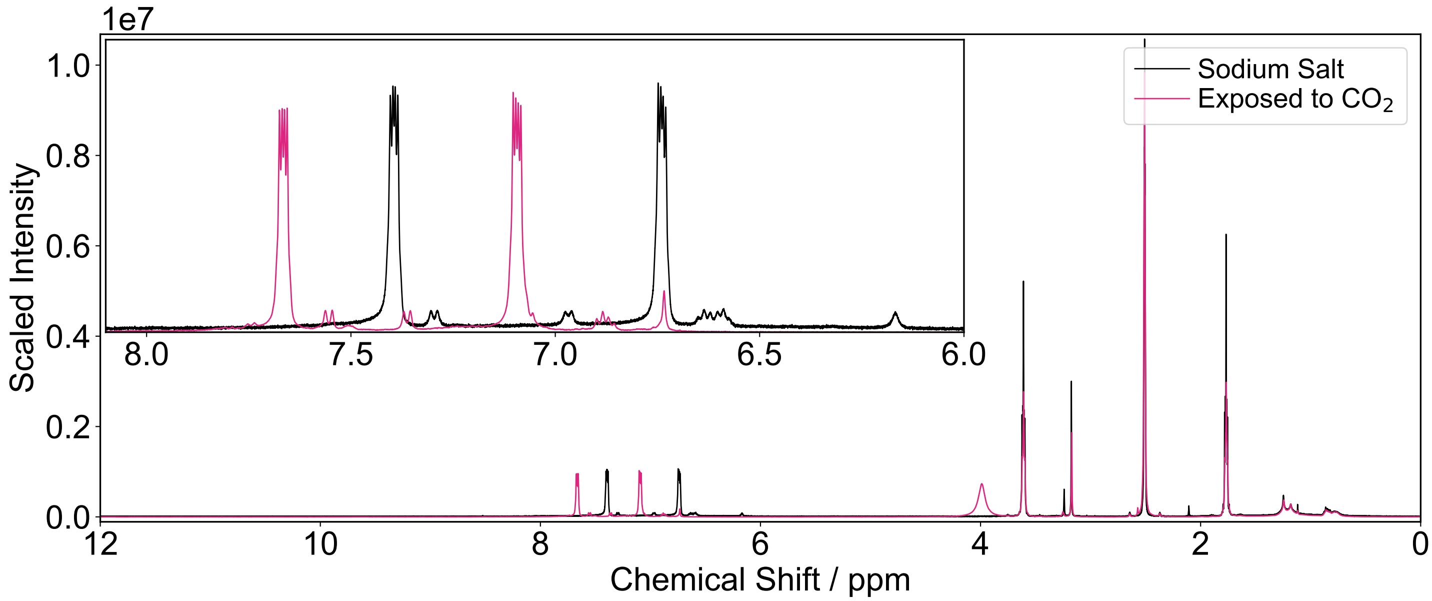

Once again there is a shift in the observed peaks in the aromatic region. Notably the number of peaks (at least for the major dichloro product) remains unchanged, offering good evidence that CO_2 is captured at both oxygen sites (since no change in molecular symmetry is observed). For the monochloro product one of the triplet peaks appears to be missing, however we believe that this is signal is masked by the dichloro peak at 7.09 ppm. This hypothesis is also supported by the integration values. The left shoulder of the missing triplet may explain the small peak at 7.06 ppm, and from the coupling constant, we therefore assign the missing triplet with a value of 7.07 ppm.

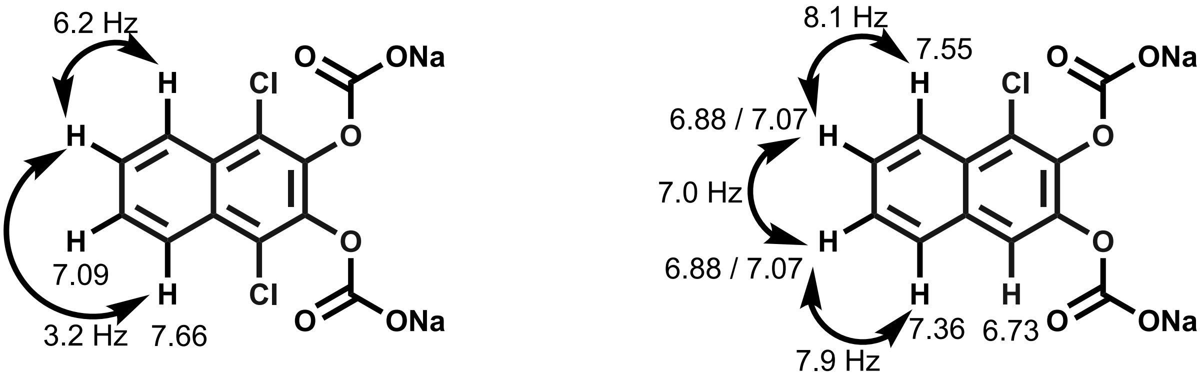

S11.4 Comparison of the Three Synthesised Reduced Species of Dichloro-2,3-Napthoquinone

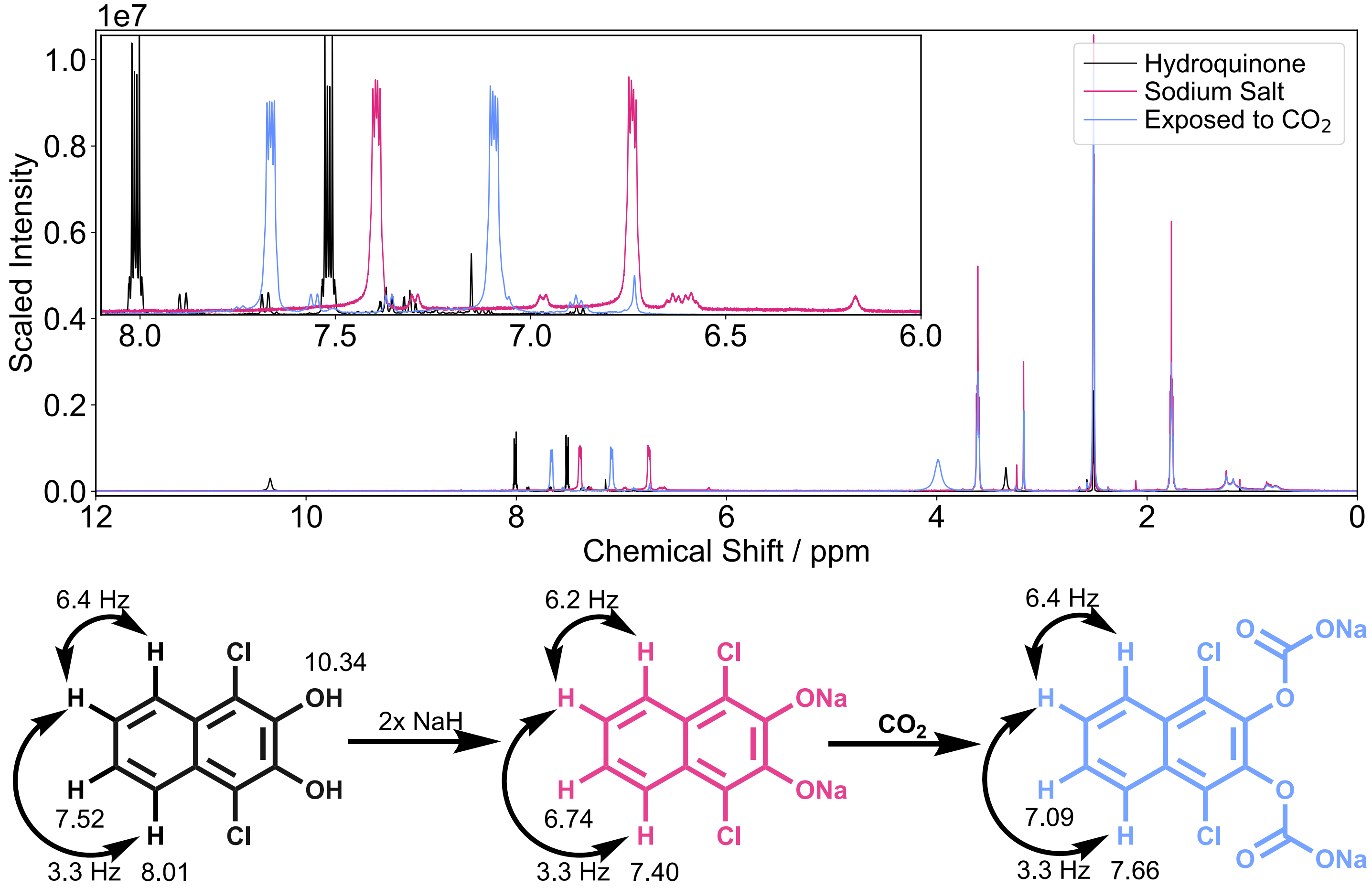

The zoom in of the aromatic region clearly shows that all three species of the major product are distinct and easily discernible. The change in peak position upon deprotonation and subsequent reaction with CO_2 is qualitatively consistent with the observations made for the non-chlorinated version and the retention in symmetry indicates that two equivalents of CO_2 are captured per quinone.

S11.5 Oxidation of the Dichlorinated Hydroquinone

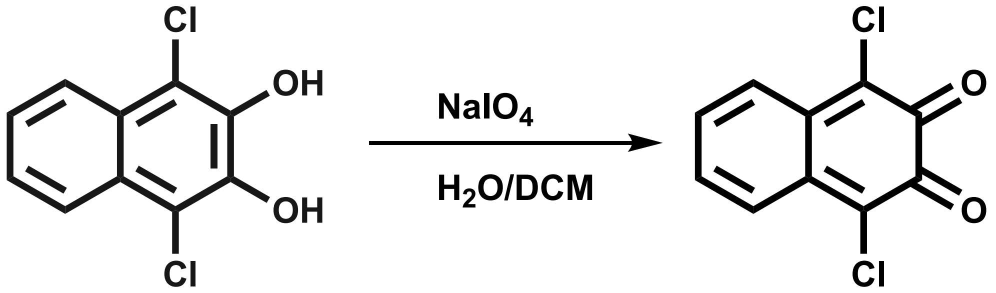

The mono-dichloro mixed product (80 mg, 0.353 mmol) was dissolved in DCM (10 mL) to form a golden orange solution. A sodium acetate buffer was made from glacial acetic acid (19 mg, 0.32 mmol) and sodium acetate (56 mg, 0.68 mmol) in deionised water (10 mL). Sodium metaperiodate (111 mg, 0.519 mmol, 1.5 eq) was dissolved in the buffer solution under N_2 at 0°C with stirring. The hydroquinone DCM solution was then added slowly to the periodate solution slowly, under N_2, at 0°C with vigorous stirring, immediately a red colour emerged as well as a precipitate. The product was extracted with an additional 35 mL of DCM and the aqueous layer with white precipitate discarded. The organic layer was dried over anhydrous magnesium sulfate, filtered, and the solvent removed *in vacuo* to yield 83 mg of red powder. 10 mg was dissolved in D_6−DMSO for NMR analysis.

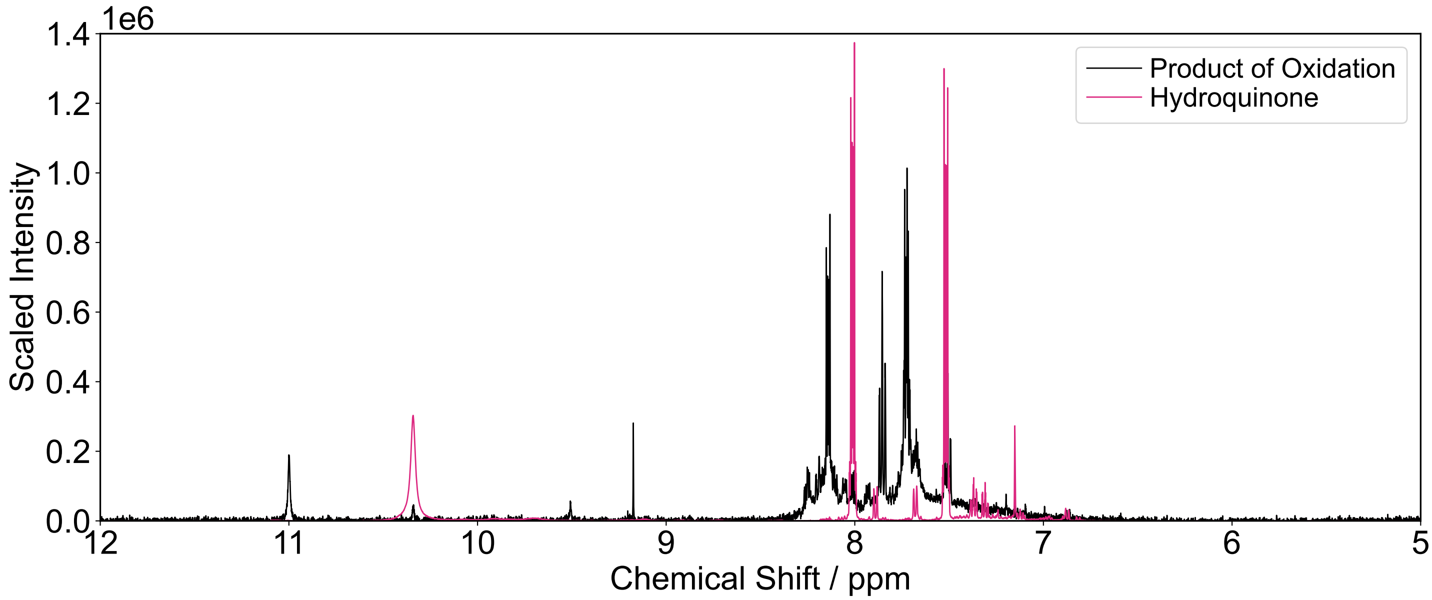

The presence of additional prominent peaks in the aromatic region, as well as the appearance of what could reasonably be hydroxy peaks (9-11 ppm), indicate that the simple oxidised form of the hydroquinone has not been isolated. It is possible that upon oxidation, reactivity towards nucleophiles such as water increases, leading to attack at the non-functionalised groups, hence the appearance of possible hydroxy groups.

Although the spectra does not indicate that the desired product was isolated, it is an improvement over the spectra obtained after the attempted oxidation of the unfunctionalised hydroquinone, Fig. S24, where only a very broad peak is seen encompassing the aromatic region. Also unlike the unfunctionalised version, this oxidised product is soluble in organic solvents. This possibly indicates that the unfunctionalised version went through a polymerisation process whereas the chlorinated version does not.

S11.6 Electrochemistry of the 1,4-Dichlor-2,3-Naphthoquinone Dianion

As well as the sodium salt of the dianion, and the oxidised product, the remainder of the dichlorinated hydroquinone was reacted with 2 equivalents of tetrabutylammonium hydroxide in methanol to generate the TBA^+ salt of the dianion, with the methanol removed via evaporation under N_2 followed by drying under vacuum.

CVs of all three products were recorded under N_2 with $TBAPF_6$ as the counter elctrolyte, Fig. S27. Unfortunately, as with the non-chlorinated version, no reversible electrochemistry was observed.

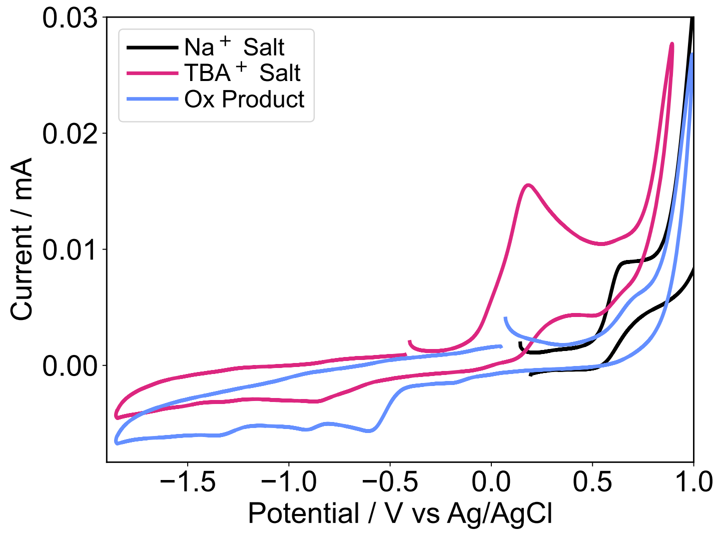

Figure S27: CVs of the sodium-salt of dichloro-2,3-naphthoquinone dianion (black), the TBA^+ salt (pink) and the product obtained from the attempted oxidation of the hydroquinone (blue). Scan rate 20 mV s^{-1}, under N_2.

Interestingly the oxidation peak observed for the TBA^+ salt is particularly well-defined and much clearer than that observed for the non-chlorinated version.

It is also observed that the oxidised product exhibits a few reduction waves, however none clearly correspond to the oxidation wave observed for either of the dianion salts. This is further evidence that the oxidised product truly is not the desired quinone.

S11.7 Discussion on the Stability of 2,3-Naphthoquinone

From the attempts to oxidise the hydroquinones, and the observed electrochemistry, it appears that functionalising at the sites *ortho* to the carbonyl groups has slightly increased the stability, however not enough to obtain good reversible electrochemistry. It is possible that the remaining non-functionalised sites remain suitably reactive, such that the oxidised quinone is not stable in the conditions explored. With further functionalisation it may be possible to reduce the reactivity to the point where stability is achieved, however further synthetic studies are out of the scope of this project.

These stability issues also highlight a fundamental flaw in modelling molecules as being completely isolated in implicit solvents. A future study on obtaining a better understanding of the decomposition pathways of *ortho* quinones would be worthwhile for advancing this field.

References

[1] P.W. Atkins. *Physical Chemistry.* Oxford University Press, 1998.

[2] Axel D Becke. "Density-functional thermochemistry. III. The role of exact exchange". In: *The Journal of chemical physics* 98.7 (1993), pp. 5648–5652.

[3] Chengteh Lee, Weitao Yang, and Robert G Parr. "Development of the Colle-Salvetti correlation-energy formula into a functional of the electron density". In: *Physical review B* 37.2 (1988), p. 785.

[4] Seymour H Vosko, Leslie Wilk, and Marwan Nusair. "Accurate spin-dependent electron liquid correlation energies for local spin density calculations: a critical analysis". In: *Canadian Journal of physics* 58.8 (1980), pp. 1200–1211.

[5] Philip J Stephens et al. "Ab initio calculation of vibrational absorption and circular dichroism spectra using density functional force fields". In: *The Journal of physical chemistry* 98.45 (1994), pp. 11623–11627.

[6] Timothy Clark et al. "Efficient diffuse function-augmented basis sets for anion calculations. III. The 3-21+G basis set for first-row elements, Li-F". In: *J. Comput. Chem.* 4 (1983), pp. 294–301. DOI: 10.1002/jcc.540040303.

[7] Michelle M. Francl et al. "Self-consistent molecular orbital methods. XXIII. A polarization-type basis set for second-row elements". In: *J. Chem. Phys.* 77 (1982), pp. 3654–3665. DOI: 10.1063/1.444267.

[8] R. Krishnan et al. "Self-consistent molecular orbital methods. XX. A basis set for correlated wave functions". In: *J. Chem. Phys.* 72 (1980), pp. 650–654. DOI: 10.1063/1.438955.

[9] A. D. McLean and G. S. Chandler. "Contracted Gaussian basis sets for molecular calculations. I. Second row atoms, Z=11-18". In: *J. Chem. Phys.* 72 (1980), pp. 5639–5648. DOI: 10.1063/1.438980.

[10] Günther W. Spitznagel et al. "An evaluation of the performance of diffuse function-augmented basis sets for second row elements, Na-Cl". In: *J. Comput. Chem.* 8 (1987), pp. 1109–1116. DOI: 10.1002/jcc.540080807.

[11] Larry A. Curtiss et al. "Extension of Gaussian-2 theory to molecules containing third-row atoms Ga-Kr". In: *J. Chem. Phys.* 103 (1995), pp. 6104–6113. DOI: 10.1063/1.470438.

[12] Vincenzo Barone and Maurizio Cossi. "Quantum calculation of molecular energies and energy gradients in solution by a conductor solvent model". In: *The Journal of Physical Chemistry A* 102.11 (1998), pp. 1995–2001.

[13] Anna T Bui et al. "Trade-off between redox potential and the strength of electrochemical CO2 capture in quinones". In: *The Journal of Physical Chemistry C* 126.33 (2022), pp. 14163–14172.

[14] Evgeny Epifanovsky et al. "Software for the frontiers of quantum chemistry: An overview of developments in the Q-Chem 5 package". In: *The Journal of chemical physics* 155.8 (2021).

[15] Xin Yin, Jing Wang, and Shufeng Shen. "Solubility of N2O and CO2 in Dimethyl Sulfoxide, 2-(Butylamino) ethanol, and Their Water-Lean Blends". In: *Journal of Chemical & Engineering Data* 69.4 (2024), pp. 1668–1676.

[16] Noémie Elgrishi et al. "A practical beginner's guide to cyclic voltammetry". In: *Journal of chemical education* 95.2 (2018), pp. 197–206.

[17] Luca Valgimigli et al. "Formation of a Blue Adduct between 4-tert-Butyl-1, 2-benzoquinone and 4-Amino-N, N-diethylaniline". In: *Tetrahedron* 56.4 (2000), pp. 659–662.

[18] Meng Yao Zhang and Russell A Barrow. "Accessing polyoxygenated dibenzofurans via the union of phenols and o-benzoquinones: rapid syntheses of metabolites isolated from ribes takare". In: *Organic letters* 19.9 (2017), pp. 2302–2305.
